# An alternative *CYB5A* transcript is expressed in aneuploid ALL and enriched in relapse

**DOI:** 10.1186/s12863-022-01041-1

**Published:** 2022-04-18

**Authors:** Lorenz Bartsch, Michael P. Schroeder, Sonja Hänzelmann, Lorenz Bastian, Juan Lázaro-Navarro, Cornelia Schlee, Jutta Ortiz Tanchez, Veronika Schulze, Konstandina Isaakidis, Michael A. Rieger, Nicola Gökbuget, Cornelia Eckert, Hubert Serve, Martin Horstmann, Martin Schrappe, Monika Brüggemann, Claudia D. Baldus, Martin Neumann

**Affiliations:** 1 Department of Hematology and Oncology, Charité, University Hospital Berlin, Campus Benjamin Franklin, 12203 Berlin, Germany; 2 Research Institute Children’s Cancer Center, Department of Pediatric Hematology and Oncology, University Medical Center Hamburg, 20251 Hamburg, Germany; 3 German Cancer Research Center (DKFZ), 69120 Heidelberg, Germany; 4 German Cancer Consortium (DKTK), 69120 Heidelberg, Germany; 5 Department of Hematology and Oncology, University Hospital Schleswig-Holstein, Campus Kiel, 24105 Kiel, Germany; 6 Department of Pediatric Hematology/Oncology, Charité, University Hospital Berlin, Campus Rudolf Virchow, 13353 Berlin, Germany; 7 Core Unit Genomics, Berlin Institute of Health, 13353 Berlin, Germany; 8 Department of Medicine, Department of Hematology/Oncology, Goethe University Hospital, 60590 Frankfurt/M, Germany; 9 Frankfurt Cancer Institute, 60590 Frankfurt/M, Germany; 10 Department of Pediatrics, University Hospital Schleswig-Holstein, Campus Kiel, 24105 Kiel, Germany

**Keywords:** B-cell precursor acute lymphoblastic leukemia, Relapse, NH, HeH, High hyperdiploid, Cryptic transcription start site, Alternative transcript, *CYB5A*, Venetoclax, Resistance mechanism

## Abstract

**Background:**

B-cell precursor acute lymphoblastic leukemia (BCP-ALL) is a genetically heterogenous malignancy with poor prognosis in relapsed adult patients. The genetic basis for relapse in aneuploid subtypes such as near haploid (NH) and high hyperdiploid (HeH) BCP-ALL is only poorly understood. Pathogenic genetic alterations remain to be identified. To this end, we investigated the dynamics of genetic alterations in a matched initial diagnosis-relapse (ID-REL) BCP-ALL cohort. Here, we firstly report the identification of the novel genetic alteration *CYB5Aalt*, an alternative transcript of *CYB5A*, in two independent cohorts.

**Methods:**

We identified *CYB5alt* in the RNAseq-analysis of a matched ID-REL BCP-ALL cohort with 50 patients and quantified its expression in various molecular BCP-ALL subtypes. Findings were validated in an independent cohort of 140 first diagnosis samples from adult BCP-ALL patients. Derived from patient material, the alternative open reading frame of *CYB5Aalt* was cloned (*pCYB5Aalt*) and *pCYB5Aalt* or the empty vector were stably overexpressed in NALM-6 cells. RNA sequencing was performed of *pCYB5Aalt* clones and empty vector controls followed by differential expression analysis, gene set enrichment analysis and complementing cell death and viability assays to determine functional implications of *CYB5Aalt*.

**Results:**

RNAseq data analysis revealed non-canonical exon usage of *CYB5Aalt* starting from a previously undescribed transcription start site. *CYB5Aalt* expression was increased in relapsed BCP-ALL and its occurrence was specific towards the shared gene expression cluster of NH and HeH BCP-ALL in independent cohorts. Overexpression of *pCYB5Aalt* in NALM-6 cells induced a distinct transcriptional program compared to empty vector controls with downregulation of pathways related to reported functions of *CYB5A* wildtype. Interestingly, *CYB5A* wildtype expression was decreased in *CYB5Aalt* samples in silico and in vitro. Additionally, *pCYB5Aalt* NALM-6 elicited a more resistant drug response.

**Conclusions:**

Across all age groups, *CYB5Aalt* was the most frequent secondary genetic event in relapsed NH and HeH BCP-ALL. In addition to its high subgroup specificity, *CYB5Aalt* is a novel candidate to be potentially implicated in therapy resistance in NH and HeH BCP-ALL. This is underlined by overexpressing *CYB5Aalt* providing first evidence for a functional role in BCL2-mediated apoptosis.

**Supplementary Information:**

The online version contains supplementary material available at 10.1186/s12863-022-01041-1.

## Background

B-cell precursor acute lymphoblastic leukemia (BCP-ALL) is a heterogeneous lymphoproliferative malignancy. Despite novel therapeutic strategies ranging from immunotherapies [1] to targeting mutational driver lesions, e.g. BCR-ABL1 [2], prognosis remains poor for refractory and relapsed BCP-ALL, in particular for adult patients [3].

BCP-ALL can be molecularly classified into various genetic subtypes with differences in clinical outcome and age-dependent prevalence [4]. The subtypes are defined by structural chromosomal alterations, e.g. translocations or recurrent aneuploidy patterns, with secondary events, such as sequence mutations and copy number alterations, in pathways related to epigenetic regulation, cell cycle, lymphoid differentiation, cytokine receptor, kinase and RAS signalling [5]. They show distinct mRNA expression [6] and methylation profiles [7] likely reflecting different leukemogenic mechanisms underlying each subtype.

Near Haploid (NH) ALL (24–30 chromosomes) and high hyperdiploid (HeH) ALL (51–67 chromosomes) as defined by conventional cytogenetics [8, 9] are two different subtypes with distinct clinical outcomes in childhood BCP-ALL [10–13]. They are defined by non-random patterns of chromosomal losses and gains as well as cooperating single nucleotide variants [14, 15]. Virtual karyotyping by SNParrays showed in pediatric patients that a near haploid karyotype with retained chromosomes (e.g. 10, 14, 18, 21) could also be observed in a duplicated manner, resulting in a high hyperdiploid karyotype with trisomies or tetrasomies of the otherwise retained chromosomes [16, 17]. In our adult patient cohort these duplicated karyotypes or karyotypes with the same non-random gain of chromosomes were identified by virtual karyotyping (WES; SNParrays) [18]. RNAseq analysis revealed shared gene expression profiles of near haploid and high hyperdiploid samples with a clear distinction to other subtypes across pediatric and adult subgroups [14, 18, 19] Secondary mutations in RAS-pathway genes and epigenetic regulators such as CREBBP [14, 15, 18] and a shared DNA methylation profile [18] have been observed in both subtypes. Due to these biological similarities and our RNAseq-based subtype classification, NH and HeH samples have been grouped together (NH/HeH) despite the clinical importance of differentiating between NH and HeH samples.

In NH and HeH BCP-ALL, underlying causes of chromosomal instability, i.e. *TP53* mutations [20] or aberrant RAG activity [21], have thus far not been identified. It remains unclear if aneuploidy resembles a driver event or an epiphenomenon. Additionally, the most frequent secondary mutations are only seen in a subset of patient samples and are inconsistently gained or lost at relapse [14, 22–24]. Functional studies are limited by the lack of appropriate models [25]. Thus, leukemogenesis of these subtypes is incompletely understood, and additional genetic alterations may contribute to pathogenesis and therapy resistance.

In addition to DNA-based genetic alterations, altered transcripts arising from alternative transcription start sites (TSS) may contribute to leukemogenesis in lymphoid neoplasms [26, 27]. In the present study, we describe the alternative transcript of Cytochrome B_5_ Type A (*CYB5A*), *CYB5A*alt, starting from a previously undescribed TSS. *CYB5A* wildtype (WT) is located on chromosome 18q22.3 and encodes for the 15.2 kDA hemeprotein Cytochrome B_5_, which reduces methemoglobin to ferrous hemoglobin and provides reducing equivalents in steroid biogenesis, lipid biosynthesis and to members of the cytochrome P450 system [28–31]. It is physiologically expressed in human B-lymphocyte lineage [32]. In drosophila, mutations in *CYB5A* WT homologue *dappled* cause the formation of melanotic tumors and dysregulation of hematopoiesis [33]. In humans, mutations in *CYB5A* WT cause type IV methemoglobinemia [34]; low *CYB5A* mRNA and protein expression is associated with shorter survival in pancreatic cancer [35].

We identify *CYB5Aalt* to be a frequent event and highly specific to the NH/HeH gene expression cluster in a cohort of 50 matched initial diagnosis-relapse samples. Further, its expression was increased in relapse. Specificity and frequency of *CYB5Aalt* was confirmed in a cohort of 140 BCP-ALL initial diagnosis samples from adult patients. Additionally, we present first evidence for a potential role of *CYB5Aalt* in apoptosis and viability by overexpressing *CYB5Aalt* in vitro in BCP-ALL cell line NALM-6.

## Methods

### Patient material

This manuscript extends analyses from two previously published cohorts including adult and pediatric BCP-ALL patients enrolled into trials of population-based German study cohorts (GMALL, AIOP-BFM, COALL) [18, 23].

For the exploratory cohort (Additional file 1), methylation, transcriptome and whole exome analysis has been performed by Schroeder et al. [23]. The cohort was designed to include relapsed BCP-ALL patients with paired initial diagnosis (ID) -relapse (REL) samples, solely including patients lacking driver fusion genes detected by routine clinical diagnostics (*BCR-ABL1*, *MLL* rearrangements, *ETV6-RUNX1*). Eighty-six samples had sufficient RNA material for gene fusion detection and were used in this study for the detection of *CYB5Aalt* (cohort 1, *n* = 86) and 80 of these samples had sufficient RNA quality for gene expression analysis in transcripts per million (TPM). The validation cohort, cohort 2 (*n* = 140, Additional file 2), represents a subset of a patient cohort of ID BCP-ALL samples with sufficient RNA data, previously published by Bastian et al. [18]. RNAseq pipeline algorithms for both cohorts are shown in Additional file 3.

### Transcript validation by reverse transcriptase polymerase chain reaction and sanger sequencing

After Ficoll density separation, RNA and DNA isolation of patient samples was performed following standard procedures (AllPrep, Quiagen, Hilden, Germany; Trizol, Life Technologies, Carlsbad, CA). RNA was transcribed to complimentary DNA (cDNA) using MMLV Reverse Transcriptase (Epicentre, Chicago, USA). For validation, Reverse Transcriptase Polymerase Chain Reaction (RT-PCR) on patient cDNA was performed using *CYB5Aalt*-specific primers (forward: 5’-TCCAGCTCCTACCTGTTACCTT-3’, reverse: 5’-GGAGGTGTTCAGTCCTCTGC-3’). PCR bands were extracted using QUIAquick Gel Extraction Kit (Quiagen, Hilden, Germany) and bilateral Sanger Sequencing using the *CYB5Aalt*-specific primers was performed. Geneious version 5.4.3 software (Biomatters Ltd., Auckland, NZ) was used for analysis.

### Cell lines and culture

The human cell line NALM-6 (BCP-ALL; ACC-128) was purchased from the DSMZ (Braunschweig, Germany). Cells were maintained in RPMI 1640 medium containing 25 mM HEPES, 2 mM L-glutamine, 1 mM sodium pyruvate, 100 U/ml and 100 mg/ml streptomycin (all from Merck Millipore, Darmstadt, Germany). 10% Fetal bovine serum (Linaris, Bettingen, Germany) was added to medium. The medium has not been changed for any experiment. Cell culture was routinely checked for mycoplasma contamination by PCR (Merck Millipore).

### Plasmid constructs and transfection

cDNA derived from *CYB5Aalt* positive patient RNA served as template for a standard PCR to amplify the alternative open reading frame of *CYB5Aalt* (*CYB5Aalt-ORF*) using the primers 5′-GCCACCATGTCCAAAACATTCATCATTGGGGAG-3′ and 5′-TGTTCAGTCCTCTGCCATGTATAGGC-3′. The 191 base pairs (bp) PCR product was cloned via TOPO vector pCR 2.1 (Invitrogen, Karlsruhe, Germany) into vector pcDNA3.1-IRES-GFP for eukaryotic overexpression (*pCYB5Aalt*). BCP-ALL cell line NALM-6 was transfected with *pCYB5Aalt* or empty vector control (*pEmpty*) by electroporation (Neon Transfection System, Invitrogen, Basel, Switzerland). After 24 h, transfected cells were treated with neomycin (0.6 mg/ml, Merck Millipore) for 4 weeks. Cells were cloned by single cell sorting for Green Fluorescent Protein (GFP) and kept in culture for additional 4 weeks. Stable integration of *pCYB5Aalt* was confirmed by standard PCR on genomic DNA, expression was confirmed by RT-PCR using cDNA of clones and *Glyceraldehyde-3-phosphate-dehydrogenase* (*GAPDH*) as internal control. Before conduction of experiments, cells were thawed, cultured for 4 weeks and expression was reconfirmed by RT-PCR.

### RNA extraction and quantitative real-time PCR

RNA of *pCYB5Aalt-* and *pEmpty*-NALM-6 clones was isolated using the Rneasy Kit (Quiagen, Hilden, Germany) and cDNA was synthesized using MMLV Reverse Transcriptase (Epicentre, Chicago, USA). Quantitative real-time PCR (qRT-PCR) was performed using the Sybr Green PCR assay (Invitrogen, Karlsruhe, Germany) following the instructor’s manual. *CYB5A* mRNA expression was measured using *CYB5A-* forward primer 5′-TGAGGATGTCGGGCACTCTA-3‘and *CYB5A*-reverse primer 5′-GAGGTGTTCAGTCCTCTGCC -3′. *GAPDH* was used as internal control (forward: 5′-GAGTCAACGGATTTGGTCGT-3′, reverse: 5′-GATCTCGCTCCTGGAAGATG-3′). Relative expression values were indicated as Δ CT value (*GAPDH*_CT_-*CYB5A*_CT_).

### WST-1 viability assays

*pCYB5Aalt* NALM-6 and empty vector controls were treated with Venetoclax (SelleckChem, München, Germany) and seeded in quintets for each concentration of Venetoclax (0, 0.2, 0.5, 1, 5, 10 μM). 1 × 10^5^ cells per well were seeded in 90 μl medium and incubated at 37 °C, 5% CO_2_. After 72 h, 10 μl of WST-1 reagent was added to each well and cells were incubated at same conditions for 3 h to allow for reduction of WST-1 to formazan by the electron transport chain of viable cells. Then, absorbance was measured using a Sunrise microplate absorbance reader (Tecan, Männerdorf, Switzerland) at 450 nm with a reference wavelength of 620 nm. Raw absorbance measurements were normalised to untreated control samples, after subtracting WST-1 absorbance measurements in wells only containing medium.

### Cell death assays

*pCYB5Aalt* NALM-6 and empty vector controls were treated with Venetoclax and seeded in duplicates for each concentration of Venetoclax (0, 0.2, 0.5, 1, 5, 10, 20 μM). 2.5 × 10^5^ cells per well were seeded in 500 μl medium and incubated at 37 °C, 5%CO_2_. After 48 h, 300 μl of cells per well were transformed to FACS tubes and washed with 4 °C Phosphate Buffered Saline. Working on ice, 50 μl Annexin V binding buffer (1:10 dilution) (BD Pharmingen, Heidelberg, Germany) and 1 μl Propidium Iodide (PI) (BD Pharmingen, Heidelberg, Germany) were added to each tube. Cells were then incubated for 15 min at room temperature in the dark. Dead cells were then analyzed for PI positivity and GFP negativity by fluorescence activated cytometry using FACSCalibur (BD Pharmingen, Heidelberg, Germany).

### High throughput sequencing analyses

Sample preparation, sequencing and bioinformatic analysis of the matched, multiomics ID-REL BCP-ALL cohort and the validation cohort have been performed as previously published. To identify *CYB5Aalt* and to determine its mRNA expression, ‘stringtie‘package [36] and ‘defuse‘package [37] were used. To increase transcript specificity, only *CYB5A* transcripts spanning a genomic distance exceeding 120,000 bp and partial alignment to *CYB5A* were used for downstream analysis of *CYB5Aalt* expression and frequency. Only samples passing quality control by RNA-SeQC [38] were used for expression analysis. Normalized mRNA Expression was reported in TPM.

### *pCYB5Aalt* NALM-6 and empty vector controls

For RNAseq, 6 samples per lane were sequenced with an average of approximately 30 million mapped reads per sample (MMRS). All sequences were aligned to the human genome build GRCh38 [39] using STAR-aligner [40]. Samples were further processed using the DESEQ2 pipeline for differential gene expression analysis [41]. Hierarchical clustering of the 500 most variably expressed genes based on DESEQ2-regularised-logarithm transformation (rlog) gene expression values and the heatmap were constructed using the ‘pheatmap’ package [42]. Principal component analysis (PCA) was performed with rlog-normalised gene expression values using DESEQ2. Gene set enrichment analysis was performed using the ‘fgsea’ package [43]. As input for ranked gene lists, mean log_2_-foldchange of genes between *pCYB5Aalt* NALM-6 and empty vector controls was used. Gene sets “Hallmark “and “KEGG subset of canonical pathways “from the ‘misgdbr’ package were used for analysis. Data visualization has been done using ‘ggplot2’ [44].

### Statistical analysis

Subtype frequency of *CYB5Aalt* was analysed by Fisher’s exact test. Inverse correlation between *CYB5Aalt* and *CYB5A* gene expression was evaluated by performing linear regression analysis. Euclidean distance with average linkage was used for unsupervised clustering. Fisher’s exact test was used to assess enrichment of *CYB5Aalt* samples in *CYB5A* low expressers in patient cohorts. Wilcoxon rank sum test was performed to compare differences in *CYB5A* gene expression as well as expression of BH3-motif genes between *CYB5Aalt* positive and negative patient samples. Group differences in PI-uptake to evaluate cell death and group differences in WST-1 reduction to formazan to assess viability were evaluated by Mann-Whitney-U-Test. All statistical tests were both-sided. Adjustment of *p*-values for multiple comparison was performed by the Benjamini-Hochberg method.

## Results

### Transcriptomic characterisation of BCP-ALL identifies *CYB5Aalt* as novel transcript

Fifty BCP-ALL samples at initial diagnosis and relapse, 26 adult and 24 pediatric patients, lacking cytogenetic rearrangements identified by conventional diagnostics (*BCR-ABL1*, *KMT2A-AFF1*, *ETV6-RUNX1*, *TCF3-PBX1*) were analysed as exploratory cohort (cohort 1, Additional file 4) [23]. ALL patients were previously classified into molecular BCP-ALL subtypes based upon their mRNA expression and methylation profiles in addition to specific chromosomal rearrangements and mutations [23]. Twelve patients (24 samples) were allocated to the Ph-like and to the DUX4r subtype, respectively. Fourteen patients (28 samples) were characterised by an aneuploid karyotype defined by 3 or more whole chromosomes affected by loss of heterozygosity (LOH) or hyperdiploidies identified by virtual karyotyping (Additional file 5) [18]. Ten out of fourteen aneuploid patients showed gains in chromosomes 4, 14, and 21, distinct methylation and mRNA expression profiles and were defined as NH/HeH BCP-ALL. Four NH / HeH samples showed LOH of most disomic chromosomes and gains in chromosome 14 and 21 suggesting a “masked” near-haploid phenotype [17]. The remaining 4 aneuploid samples all had *TP53* mutations and a masked low hypodiploid karyotype (LH) with whole chromosomal gains in 1 and 22. Further patients were assigned to the PAX5mut (2 patients), PAX5r (1 patient), BCL2r (2 patients), ZNF384f (2 patients), MLLr (1 patient) and MEF2Dr subtype (1 patient). The remaining seven samples could not be allocated to a specific subtype.

**Fig. 1:**
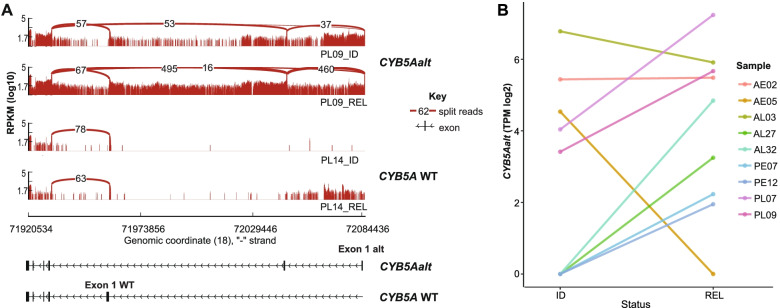
*CYB5Aalt* starts from novel TSS and *CYB5Aalt* is increased in relapse. **A** Comparison of RNAseq Read coverage (in RPKM log10) between *CYB5Aalt* positive and *CYB5A* WT samples reveals a novel TSS for *CYB5Aalt* and usage of two non-canonical exons upstream of *CYB5A* WT Exon 1 in representative patient samples (PL09 ID, PL09 REL). Arcs represent split reads between exons. Alternative Exon 2 skips *CYB5A* WT Exon 1 and splices into WT Exon 2. Exons of *CYB5Aalt* and *CYB5A* WT are illustrated beneath according to read coverage and genomic coordinates. **B*** CYB5Aalt* gene expression (in TPM log2) in matched ID-REL patient samples, which are *CYB5Aalt* positive. Colours represent matched patient samples. Connected dots represent matched expression values of patients at ID and REL

Sufficient RNA material was available for 86 out of 100 patient samples to analyse subgroup specific fusion transcripts and transcripts with non-canonical exon usage at initial diagnosis and relapse (Additional file 1). This analysis revealed a previously undescribed alternative transcript of *CYB5A*, *CYB5Aalt*, in 13 out of 86 patient samples. The alternate TSS is currently not annotated in publicly available CAGE-Seq [45] and RNAseq data [46] and thus regarded a novel finding. In antisense direction, the novel TSS of *CYB5Aalt* (hg19, chr18:72,084,437) is located 125,268 bp upstream of the wildtype TSS. RNAseq coverage and junction reads allowed for further characterisation of *CYB5Aalt* (Fig. 1A). Towards its 5’end, *CYB5Aalt* contains a novel sequence resulting in two new exons with alternative exon 2 splicing into exon 2 of *CYB5A* WT, thereby skipping exon 1 of the wildtype. Since exon 1 encodes the start of the open reading frame (ORF) of *CYB5A* WT, skipping exon 1 by *CYB5Aalt* gives rise to an alternative open reading frame, starting from an ‘ATG’-codon in exon 2 of *CYB5A* WT. This alternative ORF maintains the reading frame of the wildtype ORF and may result in a truncated version of *CYB5A* WT lacking its heme-binding domain (Additional file 6). Expression and sequence of *CYB5Aalt* was confirmed by RT-PCR followed by Sanger Sequencing (Additional file 6).

### *CYB5Aalt* frequency is increased in relapsed BCP-ALL and enriched in NH/HeH gene expression cluster

Next, *CYB5Aalt* expression was compared between initial diagnosis and relapse of matched BCP-ALL patient samples. *CYB5Aalt* expression was detected in 13 out of 86 samples (Fig. 1B). It occurred in 8 out of 43 REL samples (18.6%) and in 5 out of 43 ID samples (11.6%). Four of five samples at ID maintained *CYB5Aalt* expression in relapse. Four samples gained *CYB5Aalt* expression at relapse without detectable expression at ID. Summarised in Table 1, median bone marrow blast count of *CYB5Aalt-*positive patients (*n* = 9) was 88%. *CYB5Aalt*-negative patients (*n* = 41) had a median blast count of 92%. *CYB5Aalt* was detected in 7/37 adult and 6/49 pediatric samples (5/19 adult and 4/25 pediatric patients respectively). *CYB5Aalt* was identified in 11 out of 18 NH/HeH samples, in Ph-like (1/20) and LH (1/6) samples. Overall, the frequency of *CYB5Aalt* samples was 61.1% (11/18) in NH/HeH samples compared to 2.7% (2/68) in other BCP-ALL samples (*p* = 2 × 10^− 4^, two tailed Fisher’s exact test). Within the NH/HeH gene expression cluster, four *CYB5Aalt*-positive samples showed a masked near-haploid phenotype (4/6) and seven samples were hyperdiploid (7/12).

**Table 1:** Basic clinical characteristics and subgroup frequencies of *CYB5Aalt* positive and negative patients in cohort 1

	***CYB5Aalt-status***	
	positive (***n*** = 9)	negative (***n*** = 41)
**median BM blast count (%) at ID**	88	92
**median age at ID (years)**	18	15
**median time to REL (days)**	615	744
**samples**		
adult (*n* = 24)	5	19
pediatric (*n* = 26)	4	22
**sex**		
male (*n* = 27)	4	23
female (*n* = 23)	5	18
**molecular subgroup**		
NH (*n* = 4)	2	2
HeH (*n* = 6)	3	3
LH (*n* = 4)	1	3
Ph-like (*n* = 12)	1	11
DUX4r (*n* = 12)	0	12
Unknown (*n* = 3)	0	3
BCL2r (*n* = 2)	0	2
PAX5mut (*n* = 2)	0	2
PAX5r (*n* = 1)	0	1
MEF2Dr (*n* = 1)	0	1
MLLr (*n* = 2)	0	2
ZNF384f (*n* = 2)	0	2

Table 1 lists a comparison of characteristics for CYB5Aalt positive patients (*n* = 9) and negative patients (*n* = 41) in cohort 1 (*n* = 50). Characteristics include median bone marrow (BM) blast count, age, sex, frequencies of adult (*n* = 24) and pediatric patients (*n* = 26) and median time to REL. Further, frequencies of CYB5Aalt-positive and negative samples in the different molecular subtypes in cohort 1 are listed with total number of patients per subgroup in brackets, respectively

The specificity towards the NH/HeH gene expression cluster was further validated in an independent validation cohort of 140 BCP-ALL RNA ID patient samples, analysed by RNAseq [18], including 15 molecular subtypes including recurrent cytogenetic rearrangements identified by conventional diagnostics (*BCR-ABL1*, *KMT2A-AFF1*, *ETV6-RUNX1*, *TCF3-PBX1*) (Additional file 8). *CYB5Aalt* expression could be detected in 9 samples. 80% (4/5) NH/HeH cases were positive for *CYB5Aalt* compared to 3.7% (5/135) of non-NH / HeH samples (*p* = 3.5 × 10^− 5^, two tailed Fisher’s exact test). The five *CYB5Aalt* cases, that were not assigned to NH/HeH BCP ALL, comprised four Ph-like (*n* = 27) and one sample that could not be categorised by a subtype (*n* = 17). Although *CYB5Aalt* was not exclusively identified in NH/HeH samples, we observed statistically significant enrichment for *CYB5Aalt* in NH/HeH samples in both cohorts. *CYB5Aalt*-expression across molecular subtypes is shown in Additional file 9.

### *CYB5A* WT mRNA expression is decreased in *CYB5Aalt* samples

**Fig. 2:**
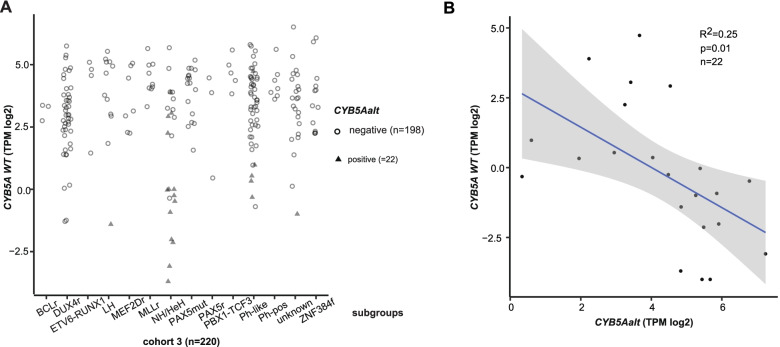
*CYB5A* WT transcript is decreased in *CYB5Aalt*-samples and shows an inverse correlation with *CYB5Aalt* mRNA expression. **A*** CYB5A* WT gene expression (TPM log2) in combined cohort 3 (*n* = 220) is shown across molecular subgroups. Circles resemble *CYB5Aalt* negative samples (*n* = 198), triangles resemble *CYB5Aalt* positive samples (*n* = 22). Subgroups with *CYB5Aalt* positive samples include NH/HeH (15/25), Ph-like (5/48), LH (1/12) and unknown (1/26). **B** Relation between *CYB5A* and *CYB5Aalt* gene expression (both in TPM log2) in *CYB5Aalt* positive samples (*n* = 22). Blue line represents linear regression line, grey area indicates 95% confidence interval

To examine the impact of *CYB5Aalt* on *CYB5A* WT expression, gene expression analysis was performed. In cohort 1, 80 samples passed quality control (Additional file 1) and mean *CYB5A* WT gene expression was significantly lower in *CYB5Aalt* samples (0.29 vs. 3.13 TPM log2, *p* = 5.19 × 10^− 5^, Wilcoxon rank sum test). In cohort 2 (*n* = 140), mean *CYB5A* WT expression was also lower in *CYB5Aalt* samples (0.004 vs. 3.759 TPM log2, *p* = 6.1 × 10^− 5^, Wilcoxon rank sum test). A total of 220 samples passed quality control and were used for expression analysis (combined cohort 3, *n* = 220). In combined cohort 3, mean gene expression of *CYB5A* WT in *CYB5Aalt* positive samples was 0.17 TPM log2 and 3.54 TPM log2 in *CYB5Aalt* negative samples (*p* = 2.1 × 10^− 9^, Wilcoxon rank sum test). *CYB5A* WT expression was mostly stable across molecular subtypes in combined cohort 3 apart from *CYB5A*alt positive samples (Fig. 2A). *CYB5Aalt* samples were highly overrepresented among the 10% of samples with the lowest *CYB5A* WT expression (68.2%, 15/22) in comparison with the rest of the cohort (3.5%, 7/198, *p* = 2.23 × 10^− 13^, two-tailed Fisher’s exact test). Further, an inverse relation between *CYB5A* WT and *CYB5Aalt* gene expression was observed (Fig. 2B) suggesting that a higher *CYB5A*alt expression contributes to a decrease in *CYB5A* WT gene expression (R^2^ = 0.25, *p* = 0.01, linear regression analysis).

### Overexpression of *CYB5Aalt*-ORF induces a distinct transcriptional program in NALM-6 cells

**Fig. 3:**
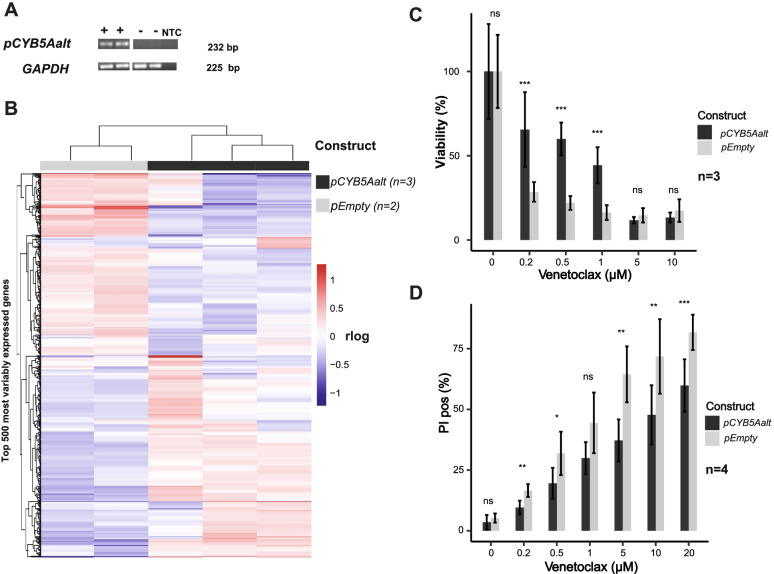
*pCYB5Aalt* Nalm 6 showed a distinct transcriptional program and resistance to Venetoclax induced cell death. **A ***CYB5Aalt-*ORF is stably overexpressed in NALM-6 cells. NALM-6 cells were transfected with *pCYB5Aalt* (+) or *pEmpty* (−). RT-PCR was used to confirm overexpression of *CYB5Aalt*-ORF mRNA. Two representative samples of each are shown. *GAPDH* was used as a control. NTC = non-template control. Full length electrophoretic gel is displayed in Additional file 17. **B ***pCYB5Aalt* NALM-6 show a distinct transcriptional program compared to empty vector controls. Samples were grouped according to the 500 most variably expressed genes between samples. Columns indicate *pCYB5Aalt* clones and empty vector controls, rows represent gene expression in rlog for each sample. Colour of the heatmap indicates relative expression strength according to the deviation from the mean of all samples in rlog. **C** Viability of *pCYB5Aalt* NALM-6 cells and *pEmpty* upon Venetoclax treatment (*n* = 3). Cells were incubated with different concentrations of Venetoclax (0 μM, 0.2 μM, 0.5 μM, 5 μM, 10 μM) for 72 h. Viability was assessed photometrically after 3 h incubation time with WST-1. Absorbance values were normalised to untreated control samples and shown as viability (%). Bars represent mean viability, error bars ±standard deviation of three independent experiments each with five technical replicates per concentration. Mann-Whitney-U-Test, ***P-value< 0.001, ns = non-significant. **D** Cell death of *pCYB5Aalt* NALM-6 and *pEmpty* upon Venetoclax treatment (*n* = 4). Cells were incubated at different concentrations of Venetoclax (0 μM, 0.2 μM, 0.5 μM, 5 μM, 10 μM, 20 μM) for 48 h. Cell death was assessed by staining cells with PI. Bars represent percentage of PI positive cells, error bars ±standard deviation of four independent experiments each with technical duplicates for each concentration. Mann-Whitney-U-Test, ***P-value< 0.001, **P-value< 0.01, *P-value< 0.05, ns = non-significant

Since *CYB5Aalt* was acquired and higher expressed in relapse, we explored its role in therapy resistance using a cell line overexpression model. *CYB5Aalt*-ORF was overexpressed in the BCP-ALL cell line NALM-6 (Fig. 3A) by stably integrating *pcDNA3.1-CYB5AaltORF-IRES-GFP* (*pCYB5Aalt)*. Similarly to *CYB5Aalt* patient samples, downregulation of *CYB5A* WT mRNA was observed in *pCYB5Aalt* NALM-6 clones compared to empty vector controls by qRT-PCR (Additional file 10).

Further, RNA sequencing was carried out for four *pCYB5Aalt* and two empty vector NALM-6 clones. Principal component analysis using the top 500 variably expressed genes showed two clusters separating empty vector clones from *pCYB5Aalt* clones (Additional file 11). One of the *pCYB5Aalt* clones clustered separately from all other clones. It was excluded from downstream analysis since this difference is most probable explained by random genomic integration of the overexpression vector and not by the expression of *CYBalt-*ORF. Using the top 500 variable expressed genes, hierarchical clustering of the genes between samples was carried out. The resulting heatmap of differentially expressed genes (Fig. 3B) showed clustering of *pCYB5Aalt* clones and empty vector controls suggesting the induction of a distinct transcriptional program by the overexpression of *CYB5Aalt*-ORF in NALM-6 cells.

To get insights into how these differences in the transcriptional program may affect biological mechanisms in *pCYB5Aalt* NALM-6, gene set enrichment analysis was carried out (Additional file 12). To explore possible involved pathways, MsigDB Hallmark and KEGG pathway gene sets comparing *pCYB5Aalt* NALM-6 and empty vector controls were used. Among significantly differentially regulated gene sets (false discovery rate (FDR) < 0.05), there were several gene sets related to the endoplasmic reticulum such as the Unfolded Protein Response (normalised enrichment score (NES) = − 1.65, FDR = 0.006), Xenobiotic metabolism (NES = − 1.57, FDR = 0.009), Peroxisome (NES = -1.82, FDR = 0.006) and Protein secretion (NES = − 1.82, FDR = 0.006). Similarly, these gene sets were significantly downregulated (FDR < 0.05) in *CYB5Aalt* patient samples compared to *CYB5A* WT samples in the NH/HeH gene expression cluster in combined cohort 3 (Additional file 13). Additionally, the DNA repair gene set was significantly downregulated (NES = − 2.05, FDR = 0.006). Cytochrome b_5_, encoded by *CYB5A* WT, is anchored in the endoplasmic reticulum membrane and the localisation domain is retained by the *CYB5Aalt*-ORF. A murine *CYB5A*^*−/−*^ model induced changes in gene and protein expression in multiple CYP-enzymes [29, 30] involved in xenobiotic metabolism. The unfolded protein stress response is interlinked with the key regulator of apoptosis BCL2 [47] suggesting a possible contribution of *CYB5Aalt* towards therapy resistance via this mechanistic link. Interestingly, the comparison of mRNA expression of genes belonging to the BCL2-family (Additional file 14) showed an upregulation of anti-apoptotic genes, e.g. *BCL2*, *BCL2L2* and *MCL1* and downregulation of pro-apoptotic genes, e.g. *BAD* and *BID,* in *pCYB5Aalt*-NALM-6. Similarly, pro-apoptotic BH3-motif genes such as *BID* (FDR = 0.01) were downregulated and anti-apoptotic genes, i.e. *BCL2L2* (FDR = 0.04) were upregulated in *CYB5Aalt*-positive samples in combined cohort 3 (Additional file 14).

### *pCYB5Aalt* Nalm-6 is more resistant to Venetoclax induced cell death

To assess, whether the observed trend towards upregulation of anti-apoptotic mediators in *pCYB5Aalt*-NALM-6 has a functional effect upon BCL2-regulated apoptosis, *pCYB5Aalt* NALM-6 and empty vector controls were incubated with the selective BCL2 inhibitor Venetoclax [48]. After 48 h, *pCYB5Aalt*-Nalm 6 and empty vector controls were stained with WST-1 as a surrogate marker for viability. *pCYB5Aalt*-Nalm 6 showed significantly (*p* < 0.05, Mann-Whitney U Test) higher viability (Fig. 3C) at different concentrations of Venetoclax (0.2 μM:0.67 vs. 0.29 absorbance at 450 nm, 0.5 μM: 0.61 vs. 0.24, 1 μM: 0.45 vs. 0.19) compared to empty vector controls in three independent experiments. Further, the cells were stained with PI to assess cell death. FACS-Analysis gating for PI positive and GFP negative cells revealed significantly (*p* < 0.05, Mann-Whitney U Test) less cell death in *pCYB5Aalt-*Nalm 6 cells at different concentrations of Venetoclax (0.2 μM: 9.6% vs. 16.6%, 0.5 μM: 19.6% vs. 31.9%, 5 μM: 37.2% vs. 64.5%, 10 μM: 47.7% vs. 71.8%, 20 μM: 47.7% vs. 71.8%) in four independent experiments (Fig. 3D).

## Discussion

The underlying mechanisms of leukemogenesis in NH and HeH BCP-ALL are incompletely understood and a mutational driver event remains to be identified. Similarly to chromophobe renal cell carcinoma and pancreatic neuroendocrine tumors [49], malignancies with a reported high fraction of unidentified drivers, a uniformly occurring aneuploidy pattern is present in NH and HeH BCP-ALL, which arises early in leukemogenesis [15, 50]. However, the functional role of this pattern as a driver or passenger event is disputed [51]. In addition, secondary mutational events are often volatile and lost or gained at relapse [15, 23]. Despite cytogenetic and outcome differences between NH and HeH BCP-ALL, they share a common gene expression and methylation profile likely reflecting a common leukemogenic mechanism with differences in clinical outcome likely dependent on a simple gain of chromosomes (HeH) or a loss of chromosomes (NH) with a possible consequential duplication of chromosomes and widespread uniparental disomies (masked-NH). Here, we add to the genetic complexity of NH and HeH BCP-ALLby introducing the alternative transcript of *CYB5A*, *CYB5Aalt*, and provide first evidence pointing towards a possible role of *CYB5Aalt* in therapy resistance. *CYB5Aalt* was firstly discovered analysing RNAseq data in a matched ID-REL cohort of 50 BCP-ALL patients (cohort 1) showing enrichment in relapse and increased occurrence in the NH/HeH gene expression cluster. In this for relapse selected cohort, *CYB5Aalt* was detected in samples with a masked near-haploid phenotype and in samples with a virtual high hyperdiploid karyotype. Relapse in HeH-BCP ALL is not common and associated with a less favourable prognosis [16, 22, 52, 53]. Interestingly, *CYB5Aalt* was the most frequent genetic event secondary to aneuploidy in relapsed HeH BCP-ALL (Additional file 15). The specificity towards the NH/HeH gene expression cluster was consequently confirmed in cohort 2, a RNAseq-cohort of 140 BCP-ALL ID patient samples and cross validated via qRT-PCR and Sanger Sequencing.

The comparison of the accompanying Exonseq data of samples positive or negative for *CYB5Aalt* did not show any differences in regional coverage suggesting that no genomic deletion detectable by exon coverage can explain de novo transcription of *CYB5Aalt*. Rather, the HeH subtype has been described to be widely hypomethylated (23) and first Hi-C experiments suggest a wide dysregulation of 3D-chromatin architecture in this subtype compared to ETV6-RUNX1 BCP-ALL [54], making epigenetic dysregulation a probable cause for this genetic event. This is further supported by enrichment for tri-methylation of H3K27 near the TSS of *CYB5Aalt* [55, 56], a histone modification frequently associated with gene silencing [57], and the incidence of comparable non-canonical exon usage starting from cryptic TSS after treating a lung cancer cell line with a combination of a histone deacetylase inhibitor and a DNA methyltransferase inhibitor [58].

To gain first insights into biological mechanisms related to BCP-ALL, that *CYB5Aalt* may influence, *pCYB5Aalt* was overexpressed in BCP-ALL cell line NALM-6. In concordance to the patient cohorts, *pCYB5Aalt* overexpression caused a decrease in expression of wildtype *CYB5A* mRNA in this functional model. Further, it induced a distinct transcriptional program in NALM-6 cells compared to empty vector controls. Gene set enrichment analysis revealed several significantly altered gene sets related to the endoplasmic reticulum, i.e. the unfolded protein stress response, xenobiotic metabolism and protein secretion as well as fatty acid metabolism and adipogenesis. These findings are in line with previously reported roles for *CYB5A* WT [28, 29, 31] and a downregulation of these pathways may be  partly explained by the observed decreased expression of wildtype *CYB5A. CYB5Aalt* leads to an alternative ORF lacking the coding sequence for the heme-binding domain, which is necessary for Cytochrome B_5_’s reducing capability [31]. This lack raises the possibility of a dominant-negative effect of *CYB5A*alt upon Cytochrome B_5_ and further studies are needed to investigate protein interactions. Interestingly, DNA repair was also significantly downregulated. Aneuploidy induces DNA damage response pathways via several proposed mechanisms. Consequent activation of p53 suppresses aneuploidy-induced tumorigenesis in mice models [59]. High hyperdiploid BCP-ALL shows prolonged metaphase which triggers a p38 and p53-mediated G1 arrest and blocks proliferation [60]. In contrast to other aneuploid BCP-ALL subtypes, e.g. hypodiploid BCP-ALL, mutations in *TP53* are no frequent event in the NH or HeH subtype. Therefore, a downregulation of DNA damage response by *CYB5Aalt* in the context of aneuploidy may contribute to a survival and proliferative advantage of leukemic cells. Further, hyperdiploidy has been associated with increased proteotoxic stress resulting in an increase in apoptosis [61]. *pCYB5Aalt* NALM-6 also show a significantly downregulated protein stress response pathway. The unfolded protein stress response controls cell fate decision via the mitochondrial pathway of apoptosis controlled by the BCL-2 protein family [47], which play a dominant role in survival of lymphoid malignancies [62]. We observed a trend towards mRNA upregulation of the anti-apoptotic *BCL2*, *MCL1*, *BCL2L1* and *BCL2L2* in *pCYB5Aalt* NALM-6 compared to empty vector controls. Notably, the same trend was observed in the combined patient cohorts (Additional file 13). To gain first insights whether BCL2-regulated apoptosis is functionally impacted in NALM-6 overexpressing *pCYB5A*alt, *pCYB5Aalt* NALM-6 and empty vector controls were incubated with Venetoclax, a specific BCL2-inhibitor, and assessed for viability and apoptosis. Although *BCL2* expression was higher in *pCYB5Aalt* NALM-6, which has been associated with higher sensitivity towards Venetoclax [63, 64], *pCYB5Aalt* NALM-6 showed both, increased viability assessed by WST-1 and reduced cell death measured by PI-staining. This may be explained by the higher expression of MCL1, which has been reported to sequester BCL2, thus preventing Venetoclax from binding BCL2 and inducing apoptosis [64–66]. The observed resistance towards Venetoclax provides first evidence for a role of *pCYB5Aalt* in mediating resistance towards BCL2-dependent apoptosis. This might be of clinical significance, although Venetoclax is still under clinical investigation as therapeutic option in refractory and relapsed (R/R) BCP-ALL [67], because preclinical data derived from patient-derived xenografts and primary BCP-ALL cells as well as a case report of three R/R T-ALL patients suggested efficacy of Venetoclax in overcoming resistance towards components of induction therapy [68–71]. Further, patients resistant to Venetoclax with chronic lymphocytic leukemia and acute myeloid leukemia, where Venetoclax has already been approved, show dismal outcomes and worse response towards standard antineoplastic therapy [72, 73]. However, further mechanistic studies are needed to gain a deeper understanding of how *CYB5Aalt* may be implicated in therapy resistance of BCP-ALL.

## Conclusion

We report the occurrence of *CYB5Aalt*, an alternative transcript of *CYB5A* arising from a previously undescribed TSS, to be highly specific for NH and HeH BCP-ALL in two independent cohorts. It is the most frequent secondary genetic event in relapsed NH and HeH BCP-ALL as assessed by RNAseq, Exonseq and gene panel sequencing analysis in a matched ID-REL cohort. Overexpressing *CYB5A*alt in BCP-ALL cell line NALM-6 provides first hints for a functional implication in BCL2-mediated apoptosis.

## Supplementary Information

**Additional file 1.** Summary table cohort 1. This table lists basic clinical characteristics (e.g. age at ID, time to REL, sex, bone marrow blast count), molecular subgroups and the status of *CYB5Aalt* for each sample of the exploratory cohort. Further, it indicates which samples had sufficient RNA to detect gene fusions and alternative transcripts, referred to as cohort 1 (*n* = 86), and passed the quality check for gene expression analysis.

**Additional file 2.** Summary table cohort 2. This table lists available basic clinical characteristics (age at ID, sex), molecular subgroup annotation and occurrence of *CYB5Aalt* for each sample of the validation cohort (cohort 2, *n* = 140). The table also shows which samples were used for detection of gene fusions (including *CYB5Aalt*) and were used for gene expression analysis.

**Additional file 3.** RNAseq pipeline algorithms of cohort 1 and cohort 2. RNAseq workflow is shown for cohort 1 and cohort 2 including most important steps of patient material collection, processing and data analysis.

**Additional file 4.** Heatmap of subgroup-specific RNA expression profiles shows subgroup specificity of *CYB5Aalt* in cohort 1. Unsupervised clustering (Euclidean, Average Linkage) of 500 most variably expressed genes is shown in samples of cohort 1 (*n* = 80). Subgroup-allocation and occurrence of *CYB5Aalt* is shown for each patient sample at the top. Columns indicate patient samples, rows represent gene expression in TPM log2 for each sample. Colour of the heatmap cells indicates relative expression strength according to the change from the mean of all samples in TPM log2.

**Additional file 5.** Virtual Karyotypes of cohort 1. Virtual karyotypes are displayed as a table. The table displays gross copy number fold changes from diploid samples and loss of heterozygosity (LOH), if whole chromosomes or major parts of a chromosome arm were affected. ICSN nomenclature has not been used, as e.g. translocations were inferred from RNAseq data but not analyzed for the construction of virtual karyotypes.

**Additional file 6.** Schematic illustration of *CYB5A* WT- and *CYB5Aalt*-transcript. The graphic summarises the differences between *CYB5A* WT (top) and *CYB5Aalt* (bottom) at a transcript level. The WT ORF (dark grey), starts in exon 1, marked by “ATG” and the arrow, is disrupted in *CYB5Aalt* due to non-canonical exon usage (dashed boxes), skipping of exon 1 WT and splicing into exon 2 of the WT. This results in an alternative ORF (light grey) starting in WT exon 2, which is exon 3 of *CYB5Aalt*, lacking the full coding sequence of the heme binding domain, shown in red. The transmembrane region (green) is contained in both transcripts.

**Additional file 7.** Validation of *CYB5Aalt* in patient samples via RT-PCR and Sanger Sequencing. **A** RT-PCR with specific primers reaching from alternative Exon 1 to Exon 2 of *CYB5A* WT confirms expression of *CYB5Aalt* in cDNA derived from two representative patient RNA samples (AL03 REL, AE02 REL). *CXCR4* expression was used as positive control, NTC = non-template control. Size of PCR product is shown in bp. Full length electrophoretic gel is shown in Additional File 16. **B** Sanger sequencing of *CYB5Aalt* PCR products in (**A**), depicted as chromatograms, confirms RNAseq sequence (top) of alternative transcript.

**Additional file 8.** Heatmap of subgroup-specific RNA expression profiles shows subgroup specificity of *CYB5Aalt* in cohort 2. Unsupervised clustering (Euclidean, Average Linkage) of 500 most variably expressed genes was performed in cohort 2 (*n* = 140). Samples could be grouped according to known subtypes of BCP-ALL (top). Further, *CYB5Aalt* occurrence is shown at the top for each patient sample. Columns indicate patient samples, rows indicate gene expression in TPM log2 for each sample. The colour of the heatmap indicates relative expression strength as deviation from the mean of all samples in TPM log2.

**Additional file 9.*** CYB5Aalt* mRNA expression across molecular subtypes in cohort 3. *CYB5Aalt* expression (TPM log2) is shown across molecular subgroups in cohort 3. Only samples with detectable *CYB5Aalt* expression (*n* = 22) are displayed. Subgroups with *CYB5Aalt* positive samples include NH/HeH (*n* = 15), Ph-like (*n* = 5), LH (*n* = 1) and unknown (*n* = 1).

**Additional file 10.** Wildtype *CYB5A* mRNA expression is lower in *pCYB5Aalt* NALM-6 than in empty vector controls. Relative mRNA expression measured by qRT-PCR is depicted as ΔCT(*GAPDH*-*CYB5A*). *GAPDH* was used as reference gene. Red dots represent mean expression of *pCYB5Aalt* NALM-6 (*n* = 11, mean = − 7.24) and empty vector controls (*n* = 3, mean = − 6.61). Red lines show standard error of the mean (*pCYB5Aalt* NALM-6: ±0.185, empty vector controls: ±0.063). The maximum and minimum expression value of *CYB5Aalt* clones were defined as outliers and excluded from statistical analysis. Mean expression was compared using both-sided t-test (*p* = 0.01).

**Additional file 11.** Sample-to-sample distance between *pCYB5Aalt* NALM-6 and Empty Vector controls by Principal Component Analysis. Principal component analysis of rlog normalised RNAseq counts from *pCYB5Aalt* Nalm 6 cells (*n* = 4) and Empty Vector controls (*n* = 2) identifies different clusters. Empty vector controls define one cluster (bottom right). Three *pCYB5Aalt* NALM-6 clones define another (top middle). The fourth *pCYB5Aalt* clone was treated as an outlier and not included in further analysis.

**Additional file 12.** Gene set enrichment analysis identifying differentially expressed pathways between *pCYB5Aalt* NALM-6 cells and Empty Vector controls. Gene set enrichment analysis was performed using the log2 foldchange/standard error of log2 foldchange between the transcriptional profiles of *pCYB5Aalt* and Empty Vector samples. **A** NES for MsigDB Hallmark pathways (*n* = 50) is displayed in a descending order, positive scores implying an upregulation. Pathways with a FDR < 0.05 are displayed in dark grey. **B** NES for MsigDB KEGG pathways (*n* = 186) is displayed in a descending order, positive score implying an upregulation. Only KEGG pathways with a FDR < 0.05 are displayed (*n* = 51).

**Additional file 13.** Gene set enrichment analysis comparing *CYB5Aalt*-positive and *CYB5A* WT samples in the NH/HeH gene expression cluster shows similar downregulated pathways to the overexpression cell line. Gene set enrichment analysis was performed between *CYB5Aalt*-positive samples (*n* = 15) and *CYB5A* WT samples (*n* = 7) in the NH/HeH cluster (*n* = 22) of combined cohort 3. NES for MsigDB Hallmark pathways (*n* = 50) is shown with positive scores implying an upregulation in *CYB5Aalt*-positive samples. Pathways with a FDR < 0.05 are displayed in dark grey. Stars indicate pathways that are also significantly downregulated (FDR < 0.05) in *pCYB5Aalt* NALM-6.

**Additional file 14.** Expression of BH3-motif genes in engineered cell lines and combined cohort 3. BH3-motif genes are subdivided according to their reported roles in apoptosis in pro-apoptotic (*BAD*, *BID*, *BCL2L11*), anti-apoptotic (*BCL2*, *BCL2L2*, *BCL2A1*) and isoform-dependent (*MCL1*, *BCL2L1*). **A** BH3-motif gene expression (in TPM) is compared between *pCYB5Aalt* NALM-6 (*n* = 3) and empty vector controls (*n* = 2). Dots represent expression of individual clones. **B** Gene expression is compared between samples positive for *CYB5Aalt* (*n* = 22) and negative samples (*n* = 198) in combined cohort 3 (*n* = 220). Dots represent mean expression; vertical lines show standard error of mean. FDR = false discovery rate (Benjamini-Hochberg method).

**Additional file 15.** Genetic alterations in relapsed NH/HeH BCP-ALL patients (*n* = 10). The table summarises the number of genetic events occurring in NH/HeH BCP-ALL patients (*n* = 10) in cohort 1 as assessed by Exonseq and gene panel sequencing. Sheet 1 (summary) summarises recurrent genetic alterations (count > 1), subdivided into gene mutations (mutational count, detected by Exonseq and panel sequencing) and copy number alterations (CNA count, Exonseq). Sheet 2 (mutations) and sheet 3 (can) list all detected genetic alterations in relapsed NH / HeH BCP-ALL patients.

**Additional file 16.** Uncropped electrophoretic gel of Additional file 7. RT-PCR, followed by Sanger Sequencing (Additional Figure 5), was performed to validate *CYB5Aalt* expression in two representative patient samples (AL03 REL, AE02 REL). **A** and **B** show the top and the bottom of the same uncropped electrophoretic gel (agarose, 1.6%) showing the expression of different genes (*CYB5A*, *CYB5Aalt*, *NTRK1*, *PEAR1*, *ZEB2*, *CXCR4*, *ZEB2*-*CXCR4*) in two patient samples (AL03 REL, AE02 REL). *CXCR4* was used as positive control. NTC = non-template-control. Ladder size is indicated in bp. Cropped parts of gel are indicated by dotted, black rectangles.

**Additional file 17.** Uncropped electrophoretic gel of Fig. 3. **A** RT-PCR was used to confirm overexpression of *pCYB5Aalt* in NALM-6 cell line. Uncropped electrophoretic gel (agarose, 1.6%) of RT-PCR results of Fig. 3 are shown. Cells were transfected with *pCYB5Aalt* (+) or *pEmpty* (−). *GAPDH* was used as control. NTC = non-template-control. Size of PCR bands are shown in bp. Cropped parts of gel that were used for Fig. 3 are indicated by dotted, black rectangles.

## Data Availability

This study extends upon analyses performed in previously published papers with RNAseq data available as stated in them [18, 23].

## Abbreviations

BCP-ALL: B-cell precursor Acute Lymphoblastic Leukemia
CYB5A: Cytochrome B_5_ Type A
TSS: Transcription Start Site
Bp: Base pairs
cDNA: Complimentary DNA
GFP: Green Fluorescent Protein
GAPDH: Glyceraldehyde-3-phosphate-dehydrogenase
qRT-PCR: Quantitative real-time Polymerase chain reaction
RT-PCR: Reverse Transcriptase Polymerase chain reaction
TPM: Transcripts per million
Rlog: Regularised logarithm transformation
WT: Wildtype
ID: Initial diagnosis
CR: Complete Remission
REL: Relapse
LOH: Loss of heterozygosity
NH / HeH: Near-haploid and high hyperdiploid
LH: Low Hypodiploid
CYB5Aalt: Alternative transcript of *CYB5A*
ORF: Open reading frame
*CYB5Aalt*-ORF: Alternative open reading frame of *CYB5Aalt*
*pCYB5Aalt*: *pcDNA3.1-CYB5AaltORF-IRES-GFP*
*pEmpty*: *pcDNA3.1-IRES-GFP*
FDR: False discovery rate
NES: Normalised enrichment score
PI: Propidium Iodide

## References

[1] Maude SL, Frey N, Shaw PA, Aplenc R, Barrett DM, Bunin NJ (2014). Chimeric antigen receptor T cells for sustained remissions in leukemia. N Engl J Med.

[2] Ottmann OG, Wassmann B, Pfeifer H, Giagounidis A, Stelljes M, Duhrsen U (2007). Imatinib compared with chemotherapy as front-line treatment of elderly patients with Philadelphia chromosome-positive acute lymphoblastic leukemia (Ph+ALL). Cancer..

[3] Gokbuget N, Dombret H, Ribera JM, Fielding AK, Advani A, Bassan R (2016). International reference analysis of outcomes in adults with B-precursor Ph-negative relapsed/refractory acute lymphoblastic leukemia. Haematologica..

[4] Iacobucci I, Mullighan CG (2017). Genetic basis of acute lymphoblastic leukemia. J Clin Oncol.

[5] Mullighan CG (2014). The genomic landscape of acute lymphoblastic leukemia in children and young adults. Hematology Am Soc Hematol Educ Program.

[6] Li JF, Dai YT, Lilljebjorn H, Shen SH, Cui BW, Bai L (2018). Transcriptional landscape of B cell precursor acute lymphoblastic leukemia based on an international study of 1,223 cases. Proc Natl Acad Sci U S A.

[7] Nordlund J, Backlin CL, Wahlberg P, Busche S, Berglund EC, Eloranta ML (2013). Genome-wide signatures of differential DNA methylation in pediatric acute lymphoblastic leukemia. Genome Biol.

[8] Safavi S, Paulsson K (2017). Near-haploid and low-hypodiploid acute lymphoblastic leukemia: two distinct subtypes with consistently poor prognosis. Blood..

[9] Paulsson K, Johansson B (2009). High hyperdiploid childhood acute lymphoblastic leukemia. Genes Chromosomes Cancer.

[10] Moorman AV, Harrison CJ, Buck GA, Richards SM, Secker-Walker LM, Martineau M (2007). Karyotype is an independent prognostic factor in adult acute lymphoblastic leukemia (ALL): analysis of cytogenetic data from patients treated on the Medical Research Council (MRC) UKALLXII/eastern cooperative oncology group (ECOG) 2993 trial. Blood..

[11] Moorman AV, Richards SM, Martineau M, Cheung KL, Robinson HM, Jalali GR (2003). Outcome heterogeneity in childhood high-hyperdiploid acute lymphoblastic leukemia. Blood..

[12] Harrison CJ, Moorman AV, Broadfield ZJ, Cheung KL, Harris RL, Reza Jalali G (2004). Three distinct subgroups of hypodiploidy in acute lymphoblastic leukaemia. Br J Haematol.

[13] Nachman JB, Heerema NA, Sather H, Camitta B, Forestier E, Harrison CJ (2007). Outcome of treatment in children with hypodiploid acute lymphoblastic leukemia. Blood..

[14] Holmfeldt L, Wei L, Diaz-Flores E, Walsh M, Zhang J, Ding L (2013). The genomic landscape of hypodiploid acute lymphoblastic leukemia. Nat Genet.

[15] Paulsson K, Lilljebjorn H, Biloglav A, Olsson L, Rissler M, Castor A (2015). The genomic landscape of high hyperdiploid childhood acute lymphoblastic leukemia. Nat Genet.

[16] Groeneveld-Krentz S, Schroeder MP, Reiter M, Pogodzinski MJ, Pimentel-Gutierrez HJ, Vagkopoulou R (2019). Aneuploidy in children with relapsed B-cell precursor acute lymphoblastic leukaemia: clinical importance of detecting a hypodiploid origin of relapse. Br J Haematol.

[17] Carroll AJ, Shago M, Mikhail FM, Raimondi SC, Hirsch BA, Loh ML (2019). Masked hypodiploidy: Hypodiploid acute lymphoblastic leukemia (ALL) mimicking hyperdiploid ALL in children: a report from the Children's oncology group. Cancer Genet.

[18] Bastian L, Schroeder MP, Eckert C, Schlee C, Tanchez JO, Kampf S (2019). PAX5 biallelic genomic alterations define a novel subgroup of B-cell precursor acute lymphoblastic leukemia. Leukemia..

[19] Gu Z, Churchman ML, Roberts KG, Moore I, Zhou X, Nakitandwe J (2019). PAX5-driven subtypes of B-progenitor acute lymphoblastic leukemia. Nat Genet.

[20] Taylor AM, Shih J, Ha G, Gao GF, Zhang X, Berger AC (2018). Genomic and functional approaches to understanding Cancer aneuploidy. Cancer Cell.

[21] Papaemmanuil E, Rapado I, Li Y, Potter NE, Wedge DC, Tubio J (2014). RAG-mediated recombination is the predominant driver of oncogenic rearrangement in ETV6-RUNX1 acute lymphoblastic leukemia. Nat Genet.

[22] Malinowska-Ozdowy K, Frech C, Schonegger A, Eckert C, Cazzaniga G, Stanulla M (2015). KRAS and CREBBP mutations: a relapse-linked malicious liaison in childhood high hyperdiploid acute lymphoblastic leukemia. Leukemia..

[23] Schroeder MP, Bastian L, Eckert C, Gokbuget N, James AR, Tanchez JO (2019). Integrated analysis of relapsed B-cell precursor acute lymphoblastic leukemia identifies subtype-specific cytokine and metabolic signatures. Sci Rep.

[24] Yang M, Safavi S, Woodward EL, Duployez N, Olsson-Arvidsson L, Ungerback J (2020). 13q12.2 deletions in acute lymphoblastic leukemia lead to upregulation of FLT3 through enhancer hijacking. Blood..

[25] Aburawi HE, Biloglav A, Johansson B, Paulsson K (2011). Cytogenetic and molecular genetic characterization of the ‘high hyperdiploid’ B-cell precursor acute lymphoblastic leukaemia cell line MHH-CALL-2 reveals a near-haploid origin. Br J Haematol.

[26] Lamprecht B, Walter K, Kreher S, Kumar R, Hummel M, Lenze D (2010). Derepression of an endogenous long terminal repeat activates the CSF1R proto-oncogene in human lymphoma. Nat Med.

[27] Zhang J, McCastlain K, Yoshihara H, Xu B, Chang Y, Churchman ML (2016). Deregulation of DUX4 and ERG in acute lymphoblastic leukemia. Nat Genet.

[28] Finn RD, McLaughlin LA, Hughes C, Song C, Henderson CJ, Roland WC (2011). Cytochrome b5 null mouse: a new model for studying inherited skin disorders and the role of unsaturated fatty acids in normal homeostasis. Transgenic Res.

[29] Henderson CJ, McLaughlin LA, Finn RD, Ronseaux S, Kapelyukh Y, Wolf CR (2014). A role for cytochrome b5 in the in vivo disposition of anticancer and cytochrome P450 probe drugs in mice. Drug Metab Dispos.

[30] McLaughlin LA, Ronseaux S, Finn RD, Henderson CJ, Roland WC (2010). Deletion of microsomal cytochrome b5 profoundly affects hepatic and extrahepatic drug metabolism. Mol Pharmacol.

[31] Schenkman JB, Jansson I (2003). The many roles of cytochrome b5. Pharmacol Ther.

[32] Novershtern N, Subramanian A, Lawton LN, Mak RH, Haining WN, McConkey ME (2011). Densely interconnected transcriptional circuits control cell states in human hematopoiesis. Cell..

[33] Kleinhesselink K, Conway C, Sholer D, Huang I, Kimbrell DA (2011). Regulation of hemocytes in Drosophila requires dappled cytochrome b5. Biochem Genet.

[34] Hegesh E, Hegesh J, Kaftory A (1986). Congenital methemoglobinemia with a deficiency of cytochrome b5. N Engl J Med.

[35] Giovannetti E, Wang Q, Avan A, Funel N, Lagerweij T, Lee JH (2014). Role of CYB5A in pancreatic cancer prognosis and autophagy modulation. J Natl Cancer Inst.

[36] Pertea M, Pertea GM, Antonescu CM, Chang TC, Mendell JT, Salzberg SL (2015). StringTie enables improved reconstruction of a transcriptome from RNA-seq reads. Nat Biotechnol.

[37] McPherson A, Hormozdiari F, Zayed A, Giuliany R, Ha G, Sun MG (2011). deFuse: an algorithm for gene fusion discovery in tumor RNA-Seq data. PLoS Comput Biol.

[38] DeLuca DS, Levin JZ, Sivachenko A, Fennell T, Nazaire MD, Williams C (2012). RNA-SeQC: RNA-seq metrics for quality control and process optimization. Bioinformatics..

[39] Church DM, Schneider VA, Graves T, Auger K, Cunningham F, Bouk N (2011). Modernizing reference genome assemblies. PLoS Biol.

[40] Dobin A, Davis CA, Schlesinger F, Drenkow J, Zaleski C, Jha S (2013). STAR: ultrafast universal RNA-seq aligner. Bioinformatics..

[41] Love MI, Huber W, Anders S (2014). Moderated estimation of fold change and dispersion for RNA-seq data with DESeq2. Genome Biol.

[42] Kolde R (2012). Pheatmap: pretty heatmaps. R Package Version 61.

[43] Subramanian A, Tamayo P, Mootha VK, Mukherjee S, Ebert BL, Gillette MA (2005). Gene set enrichment analysis: a knowledge-based approach for interpreting genome-wide expression profiles. Proc Natl Acad Sci U S A.

[44] Wickham H (2016). ggplot2: Elegeant Graphics for Data Analysis.

[45] Lizio M, Abugessaisa I, Noguchi S, Kondo A, Hasegawa A, Hon CC (2019). Update of the FANTOM web resource: expansion to provide additional transcriptome atlases. Nucleic Acids Res.

[46] Consortium GT (2020). The GTEx consortium atlas of genetic regulatory effects across human tissues. Science..

[47] Hetz C (2012). The unfolded protein response: controlling cell fate decisions under ER stress and beyond. Nat Rev Mol Cell Biol..

[48] Souers AJ, Leverson JD, Boghaert ER, Ackler SL, Catron ND, Chen J (2013). ABT-199, a potent and selective BCL-2 inhibitor, achieves antitumor activity while sparing platelets. Nat Med.

[49] Consortium ITP-CAoWG (2020). Pan-cancer analysis of whole genomes. Nature..

[50] Bateman CM, Alpar D, Ford AM, Colman SM, Wren D, Morgan M (2015). Evolutionary trajectories of hyperdiploid ALL in monozygotic twins. Leukemia..

[51] Sheltzer JM, Amon A (2011). The aneuploidy paradox: costs and benefits of an incorrect karyotype. Trends Genet.

[52] Davidsson J, Paulsson K, Lindgren D, Lilljebjorn H, Chaplin T, Forestier E (2010). Relapsed childhood high hyperdiploid acute lymphoblastic leukemia: presence of preleukemic ancestral clones and the secondary nature of microdeletions and RTK-RAS mutations. Leukemia..

[53] Inthal A, Zeitlhofer P, Zeginigg M, Morak M, Grausenburger R, Fronkova E (2012). CREBBP HAT domain mutations prevail in relapse cases of high hyperdiploid childhood acute lymphoblastic leukemia. Leukemia..

[54] Yang M, Vesterlund M, Siavelis I, Moura-Castro LH, Castor A, Fioretos T (2019). Proteogenomics and hi-C reveal transcriptional dysregulation in high hyperdiploid childhood acute lymphoblastic leukemia. Nat Commun.

[55] UCSC Genome Browser. [Available from: https://genome.ucsc.edu/s/bartschl/hg19.

[56] Rosenbloom KR, Sloan CA, Malladi VS, Dreszer TR, Learned K, Kirkup VM (2013). ENCODE data in the UCSC genome browser: year 5 update. Nucleic Acids Res.

[57] Barski A, Cuddapah S, Cui K, Roh TY, Schones DE, Wang Z (2007). High-resolution profiling of histone methylations in the human genome. Cell..

[58] Brocks D, Schmidt CR, Daskalakis M, Jang HS, Shah NM, Li D (2017). DNMT and HDAC inhibitors induce cryptic transcription start sites encoded in long terminal repeats. Nat Genet.

[59] Li M, Fang X, Baker DJ, Guo L, Gao X, Wei Z (2010). The ATM-p53 pathway suppresses aneuploidy-induced tumorigenesis. Proc Natl Acad Sci U S A.

[60] Molina O, Vinyoles M, Granada I, Roca-Ho H, Gutierrez-Aguera F, Valledor L (2020). Impaired condensin complex and Aurora B kinase underlie mitotic and chromosomal defects in hyperdiploid B-cell ALL. Blood..

[61] Santaguida S, Amon A (2015). Short- and long-term effects of chromosome mis-segregation and aneuploidy. Nat Rev Mol Cell Biol.

[62] Vaux DL, Cory S, Adams JM (1988). Bcl-2 gene promotes haemopoietic cell survival and cooperates with c-myc to immortalize pre-B cells. Nature..

[63] Seyfried F, Demir S, Horl RL, Stirnweiss FU, Ryan J, Scheffold A (2019). Prediction of venetoclax activity in precursor B-ALL by functional assessment of apoptosis signaling. Cell Death Dis.

[64] Alford SE, Kothari A, Loeff FC, Eichhorn JM, Sakurikar N, Goselink HM (2015). BH3 inhibitor sensitivity and Bcl-2 dependence in primary acute lymphoblastic leukemia cells. Cancer Res.

[65] Choudhary GS, Al-Harbi S, Mazumder S, Hill BT, Smith MR, Bodo J (2015). MCL-1 and BCL-xL-dependent resistance to the BCL-2 inhibitor ABT-199 can be overcome by preventing PI3K/AKT/mTOR activation in lymphoid malignancies. Cell Death Dis.

[66] Niu X, Zhao J, Ma J, Xie C, Edwards H, Wang G (2016). Binding of released Bim to mcl-1 is a mechanism of intrinsic resistance to ABT-199 which can be overcome by combination with Daunorubicin or Cytarabine in AML cells. Clin Cancer Res.

[67] U.S. National Library of Medicine. [Available from: https://clinicaltrials.gov/ct2/results?cond=Acute+Lymphoblastic+Leukemia&term=Venetoclax&cntry=&state=&city=&dist=. 10.1080/15360280801989377 28792816

[68] Autry RJ, Paugh SW, Carter R, Shi L, Liu J, Ferguson DC (2020). Integrative genomic analyses reveal mechanisms of glucocorticoid resistance in acute lymphoblastic leukemia. Nat Cancer.

[69] Fischer U, Forster M, Rinaldi A, Risch T, Sungalee S, Warnatz HJ (2015). Genomics and drug profiling of fatal TCF3-HLF-positive acute lymphoblastic leukemia identifies recurrent mutation patterns and therapeutic options. Nat Genet.

[70] Frismantas V, Dobay MP, Rinaldi A, Tchinda J, Dunn SH, Kunz J (2017). Ex vivo drug response profiling detects recurrent sensitivity patterns in drug-resistant acute lymphoblastic leukemia. Blood..

[71] La Starza R, Cambo B, Pierini A, Bornhauser B, Montanaro A, Bourquin JP, et al. Venetoclax and Bortezomib in relapsed/refractory early T-cell precursor acute lymphoblastic leukemia. JCO Precis Oncol. 2019;3. 10.1200/PO.19.00172 PMC7448796 32923866

[72] Eyre TA, Kirkwood AA, Gohill S, Follows G, Walewska R, Walter H (2019). Efficacy of venetoclax monotherapy in patients with relapsed chronic lymphocytic leukaemia in the post-BCR inhibitor setting: a UK wide analysis. Br J Haematol.

[73] Maiti A, Rausch CR, Cortes JE, Pemmaraju N, Daver NG, Ravandi F (2021). Outcomes of relapsed or refractory acute myeloid leukemia after frontline hypomethylating agent and venetoclax regimens. Haematologica..

