# Supplementary material for: An alternative CYB5A transcript is expressed in aneuploid ALL and enriched in relapse
Source: BMC Genom Data. 2022 Apr 18;23:30. doi: 10.1186/s12863-022-01041-1 (PMC9014596; doi:10.1186/s12863-022-01041-1)
Supplement: Supplementary file 3 — Additional file 3. RNAseq pipeline algorithms of cohort 1 and cohort 2. RNAseq workflow is shown for cohort 1 and cohort 2 including most important steps of patient material collection, processing and data analysis. [file 12863_2022_1041_MOESM3_ESM.pdf]

Cohort 1

(samples= 86)

Patient material

-lack of *BCR-ABL1*, *MLL* rearrangements, *ETV6-RUNX1*

-samples retrieved at initial diagnosis (ID), relapse (REL)

-minimal residual disease (MRD) <0.01 at remission

-22 adult patients treated according to GMALL treatment protocol

-25 pediatric patients treated according to BFM/COALL treatment protocoll

RNA isolation

-Trizol

-RNA integrity number (RIN) > 7 for sequencing

Sequencing

-Illumina HiSeq4000

-six samples per lane

-sequenced with average of 64 million mapped reads per sampel (MMRS)

Alignment

-GRCh 37.75

-STAR-aligner

Gene fusion detection

-defuse v0.7.0

-FusionCatcher v0.99.7cv beta

Expression quantification

-Stringtie

Differential gene expression

-Llimma Voom

Cohort 2

(samples= 140)

Patient material

-samples retrieved at ID

-140 adult patients treated according to GMALL treatment protocol

RNA isolation

-Trizol

-RNA integrity number (RIN) > 7 for sequencing

Sequencing

-Illumina HiSeq4000

-six samples per lane

-sequenced with average of 64 million mapped reads per sampel (MMRS)

Alignment

-GRCh 37.75

-STAR-aligner

Gene fusion detection

-defuse v0.7.0

-FusionCatcher v0.99.

Expression quantification

-Stringtie

Differential gene expression

-Limma Voom
