# Supplementary figures and images for: An alternative CYB5A transcript is expressed in aneuploid ALL and enriched in relapse
Source: BMC Genom Data. 2022 Apr 18;23:30. doi: 10.1186/s12863-022-01041-1 (PMC9014596; doi:10.1186/s12863-022-01041-1)

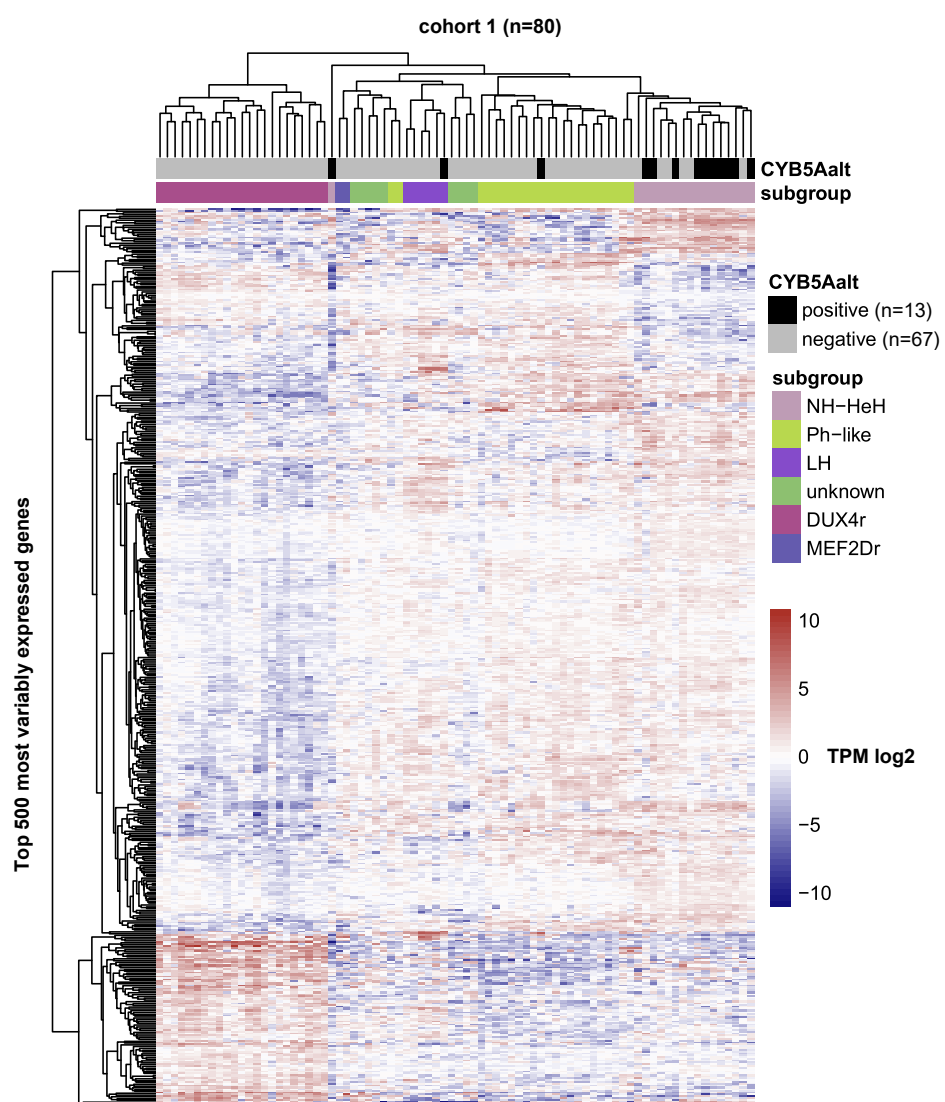

Supplement: Supplementary file 4 — Additional file 4. Heatmap of subgroup-specific RNA expression profiles shows subgroup specificity of CYB5Aalt in cohort 1. Unsupervised clustering (Euclidean, Average Linkage) of 500 most variably expressed genes is shown in samples of cohort 1 (n = 80). Subgroup-allocation and occurrence of CYB5Aalt is shown for each patient sample at the top. Columns indicate patient samples, rows represent gene expression in TPM log2 for each sample. Colour of the heatmap cells indicates relative expression strength according to the change from the mean of all samples in TPM log2. [file 12863_2022_1041_MOESM4_ESM.pdf]

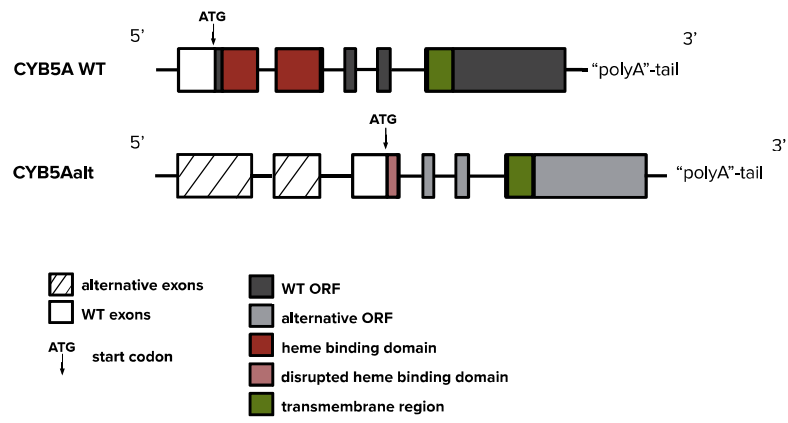

Supplement: Supplementary file 6 — Additional file 6. Schematic illustration of CYB5A WT- and CYB5Aalt-transcript. The graphic summarises the differences between CYB5A WT (top) and CYB5Aalt (bottom) at a transcript level. The WT ORF (dark grey), starts in exon 1, marked by “ATG” and the arrow, is disrupted in CYB5Aalt due to non-canonical exon usage (dashed boxes), skipping of exon 1 WT and splicing into exon 2 of the WT. This results in an alternative ORF (light grey) starting in WT exon 2, which is exon 3 of CYB5Aalt, lacking the full coding sequence of the heme binding domain, shown in red. The transmembrane region (green) is contained in both transcripts. [file 12863_2022_1041_MOESM6_ESM.pdf]

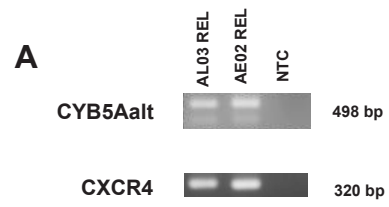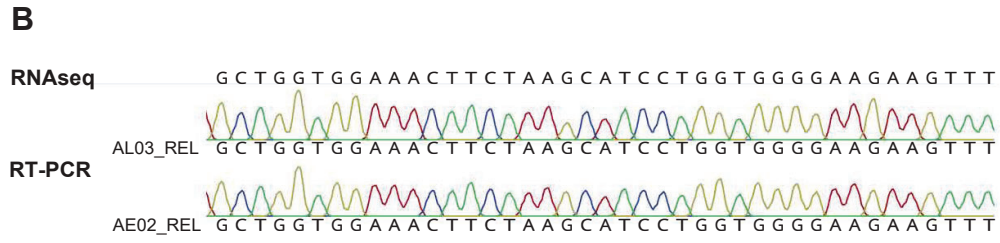

Supplement: Supplementary file 7 — Additional file 7. Validation of CYB5Aalt in patient samples via RT-PCR and Sanger Sequencing. A RT-PCR with specific primers reaching from alternative Exon 1 to Exon 2 of CYB5A WT confirms expression of CYB5Aalt in cDNA derived from two representative patient RNA samples (AL03 REL, AE02 REL). CXCR4 expression was used as positive control, NTC = non-template control. Size of PCR product is shown in bp. Full length electrophoretic gel is shown in Additional File 16. B Sanger sequencing of CYB5Aalt PCR products in (A), depicted as chromatograms, confirms RNAseq sequence (top) of alternative transcript. [file 12863_2022_1041_MOESM7_ESM.pdf]

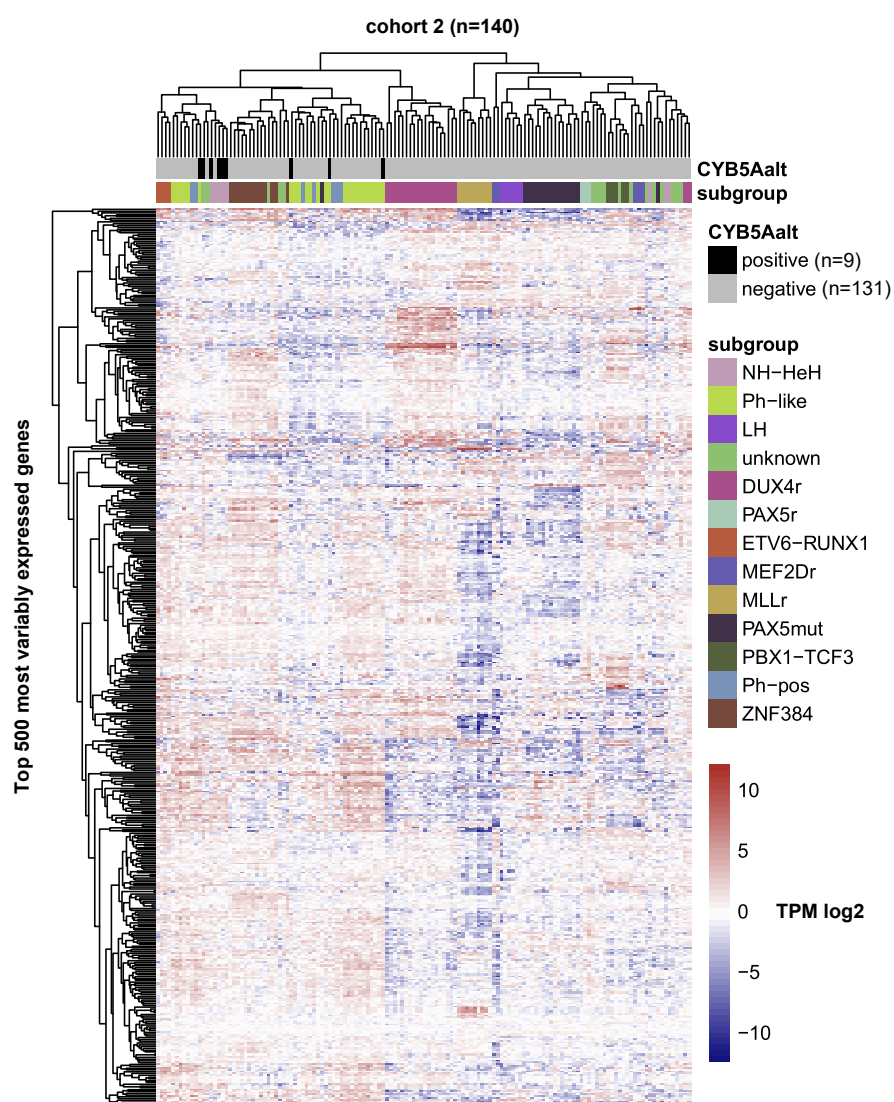

Supplement: Supplementary file 8 — Additional file 8. Heatmap of subgroup-specific RNA expression profiles shows subgroup specificity of CYB5Aalt in cohort 2. Unsupervised clustering (Euclidean, Average Linkage) of 500 most variably expressed genes was performed in cohort 2 (n = 140). Samples could be grouped according to known subtypes of BCP-ALL (top). Further, CYB5Aalt occurrence is shown at the top for each patient sample. Columns indicate patient samples, rows indicate gene expression in TPM log2 for each sample. The colour of the heatmap indicates relative expression strength as deviation from the mean of all samples in TPM log2. [file 12863_2022_1041_MOESM8_ESM.pdf]

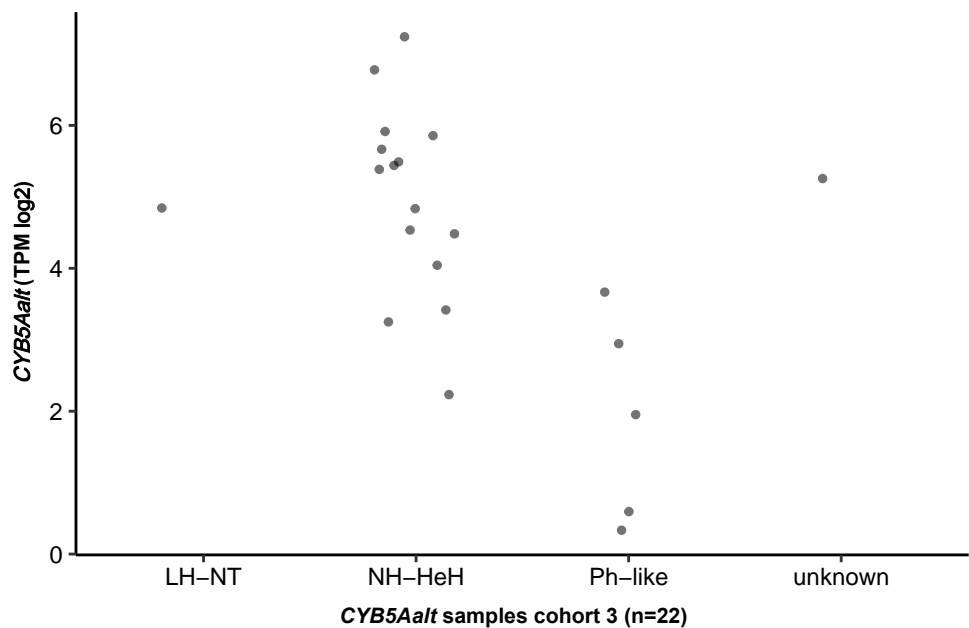

Supplement: Supplementary file 9 — Additional file 9. CYB5Aalt mRNA expression across molecular subtypes in cohort 3. CYB5Aalt expression (TPM log2) is shown across molecular subgroups in cohort 3. Only samples with detectable CYB5Aalt expression (n = 22) are displayed. Subgroups with CYB5Aalt positive samples include NH/HeH (n = 15), Ph-like (n = 5), LH (n = 1) and unknown (n = 1). [file 12863_2022_1041_MOESM9_ESM.pdf]

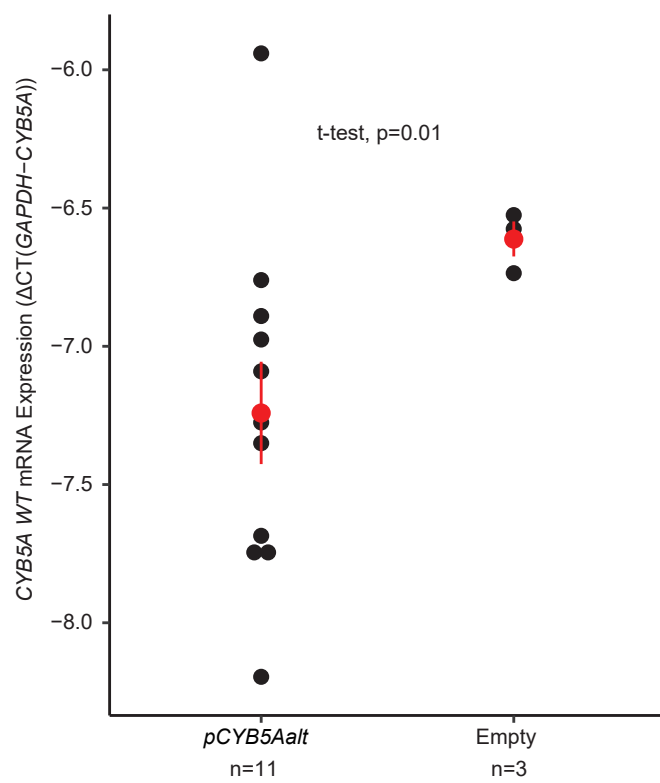

Supplement: Supplementary file 10 — Additional file 10. Wildtype CYB5A mRNA expression is lower in pCYB5Aalt NALM-6 than in empty vector controls. Relative mRNA expression measured by qRT-PCR is depicted as ΔCT(GAPDH-CYB5A). GAPDH was used as reference gene. Red dots represent mean expression of pCYB5Aalt NALM-6 (n = 11, mean = − 7.24) and empty vector controls (n = 3, mean = − 6.61). Red lines show standard error of the mean (pCYB5Aalt NALM-6: ±0.185, empty vector controls: ±0.063). The maximum and minimum expression value of CYB5Aalt clones were defined as outliers and excluded from statistical analysis. Mean expression was compared using both-sided t-test (p = 0.01). [file 12863_2022_1041_MOESM10_ESM.pdf]

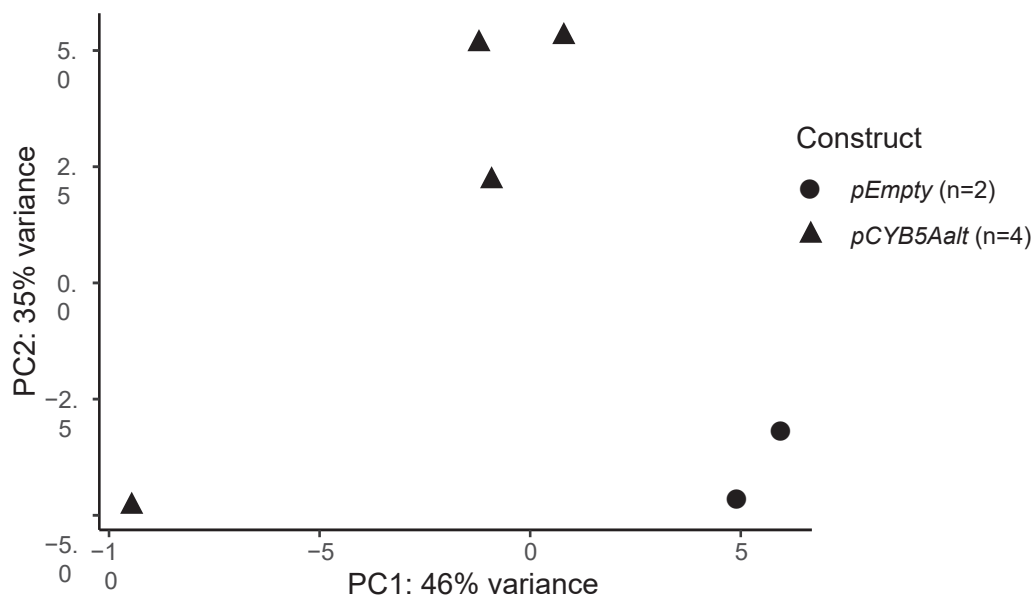

Supplement: Supplementary file 11 — Additional file 11. Sample-to-sample distance between pCYB5Aalt NALM-6 and Empty Vector controls by Principal Component Analysis. Principal component analysis of rlog normalised RNAseq counts from pCYB5Aalt Nalm 6 cells (n = 4) and Empty Vector controls (n = 2) identifies different clusters. Empty vector controls define one cluster (bottom right). Three pCYB5Aalt NALM-6 clones define another (top middle). The fourth pCYB5Aalt clone was treated as an outlier and not included in further analysis. [file 12863_2022_1041_MOESM11_ESM.pdf]

A

Hallmark pathways

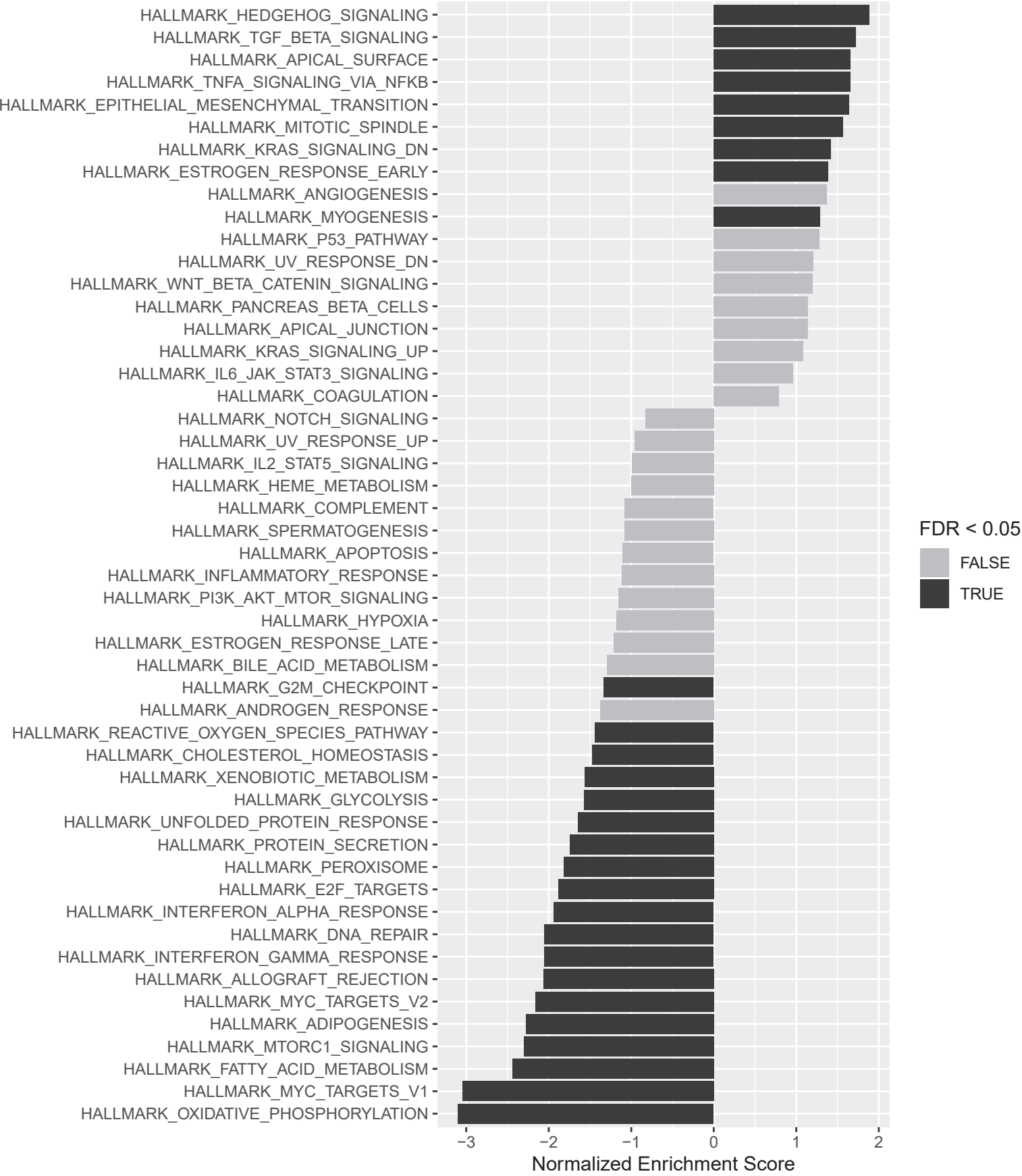

B

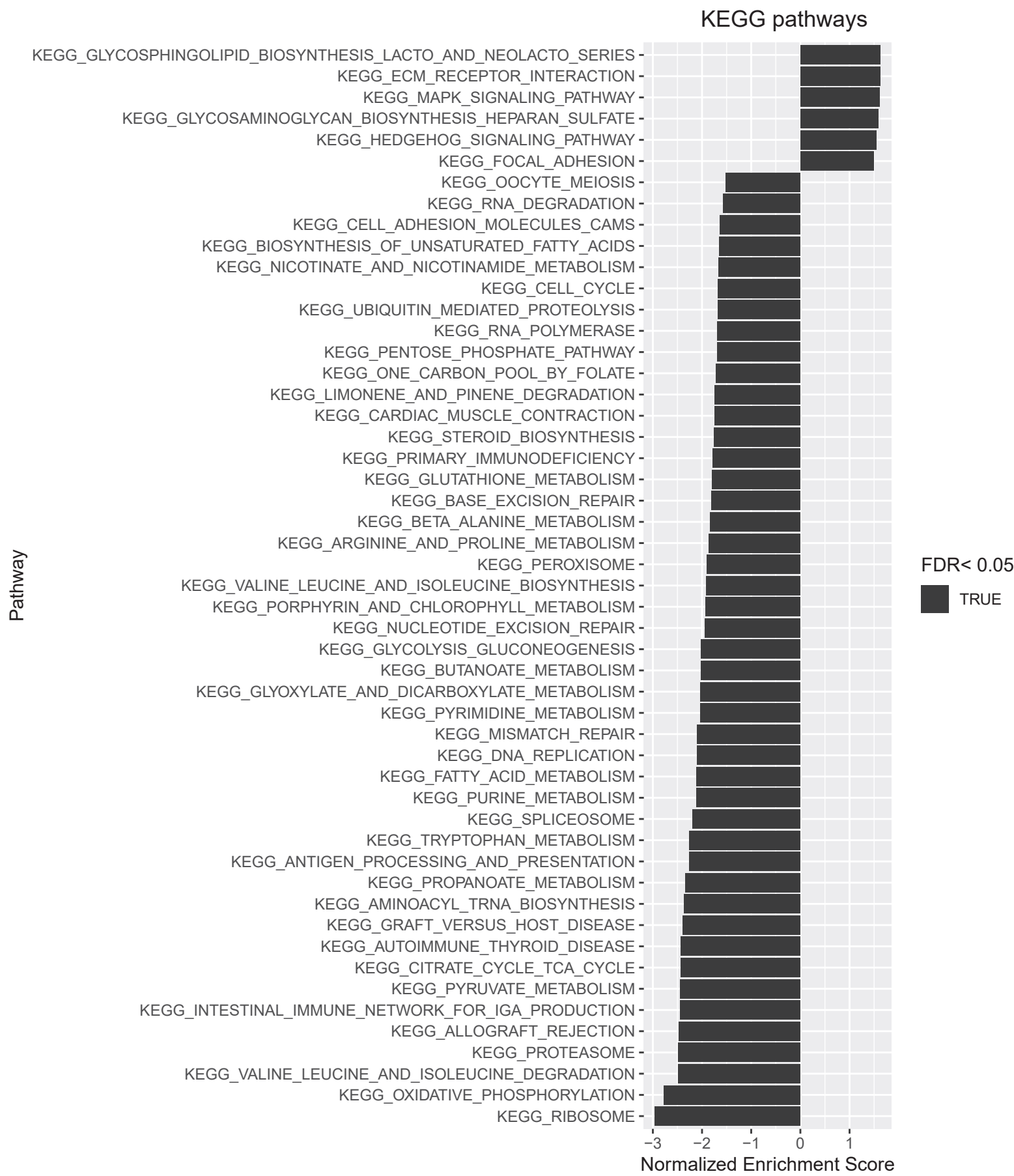

Supplement: Supplementary file 12 — Additional file 12. Gene set enrichment analysis identifying differentially expressed pathways between pCYB5Aalt NALM-6 cells and Empty Vector controls. Gene set enrichment analysis was performed using the log2 foldchange/standard error of log2 foldchange between the transcriptional profiles of pCYB5Aalt and Empty Vector samples. A NES for MsigDB Hallmark pathways (n = 50) is displayed in a descending order, positive scores implying an upregulation. Pathways with a FDR < 0.05 are displayed in dark grey. B NES for MsigDB KEGG pathways (n = 186) is displayed in a descending order, positive score implying an upregulation. Only KEGG pathways with a FDR < 0.05 are displayed (n = 51). [file 12863_2022_1041_MOESM12_ESM.pdf]

CYB5A<sup>alt</sup> vs. CYB5A WT samples in NH-HeH subtype

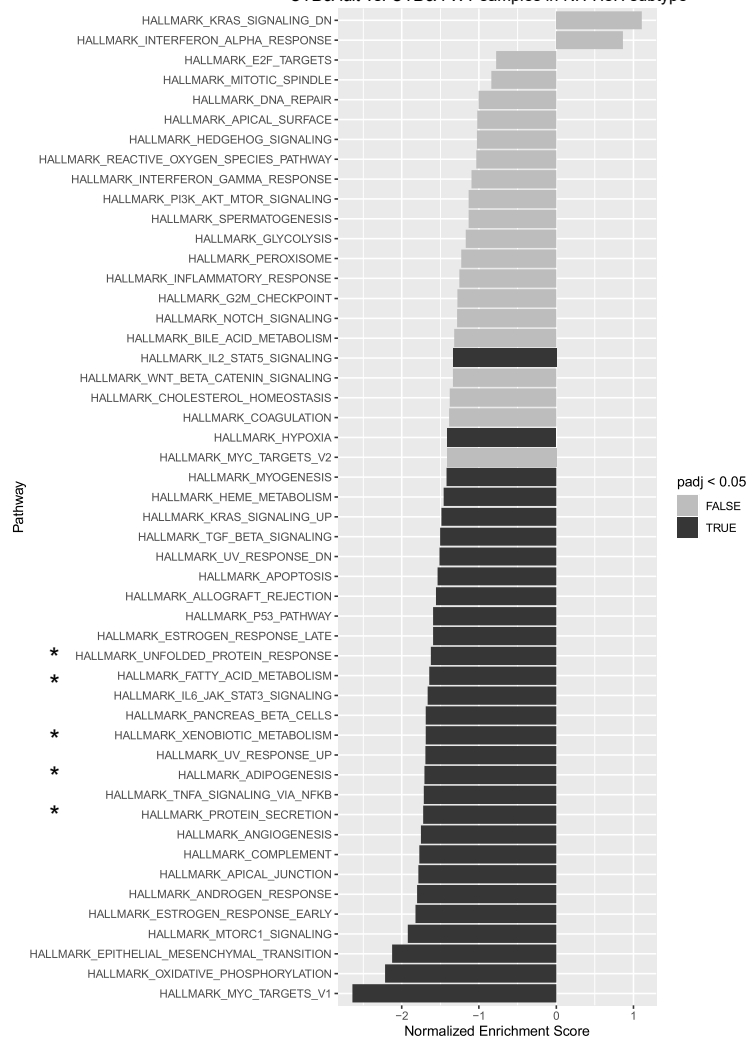

Supplement: Supplementary file 13 — Additional file 13. Gene set enrichment analysis comparing CYB5Aalt-positive and CYB5A WT samples in the NH/HeH gene expression cluster shows similar downregulated pathways to the overexpression cell line. Gene set enrichment analysis was performed between CYB5Aalt-positive samples (n = 15) and CYB5A WT samples (n = 7) in the NH/HeH cluster (n = 22) of combined cohort 3. NES for MsigDB Hallmark pathways (n = 50) is shown with positive scores implying an upregulation in CYB5Aalt-positive samples. Pathways with a FDR < 0.05 are displayed in dark grey. Stars indicate pathways that are also significantly downregulated (FDR < 0.05) in pCYB5Aalt NALM-6. [file 12863_2022_1041_MOESM13_ESM.pdf]

A

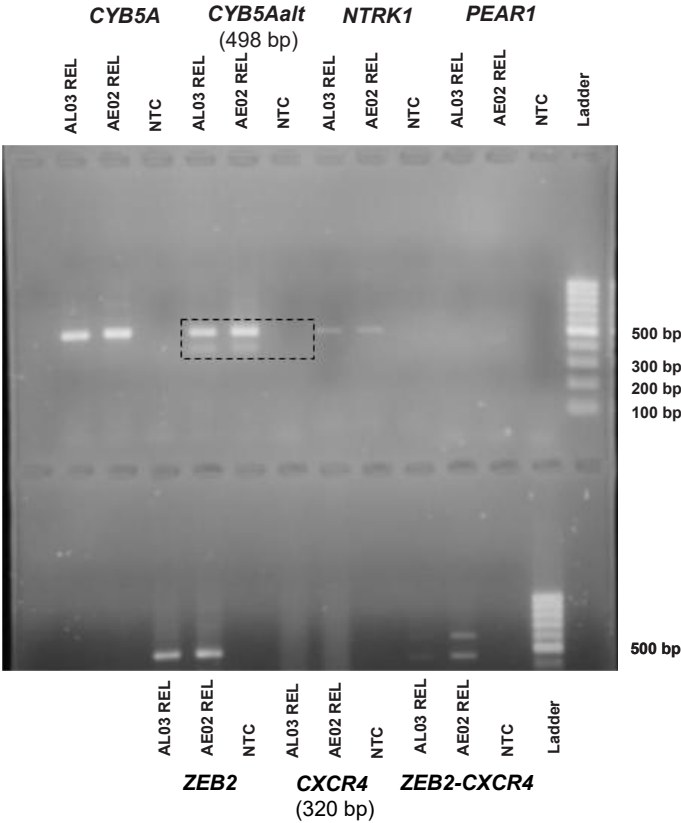

B

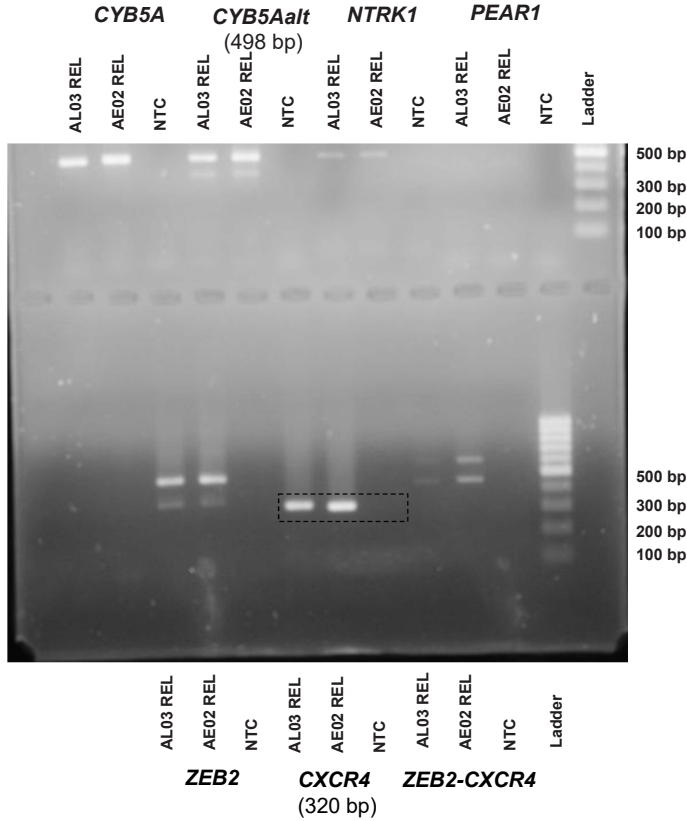

Supplement: Supplementary file 16 — Additional file 16. Uncropped electrophoretic gel of Additional file 7. RT-PCR, followed by Sanger Sequencing (Additional Figure 5), was performed to validate CYB5Aalt expression in two representative patient samples (AL03 REL, AE02 REL). A and B show the top and the bottom of the same uncropped electrophoretic gel (agarose, 1.6%) showing the expression of different genes (CYB5A, CYB5Aalt, NTRK1, PEAR1, ZEB2, CXCR4, ZEB2-CXCR4) in two patient samples (AL03 REL, AE02 REL). CXCR4 was used as positive control. NTC = non-template-control. Ladder size is indicated in bp. Cropped parts of gel are indicated by dotted, black rectangles. [file 12863_2022_1041_MOESM16_ESM.pdf]

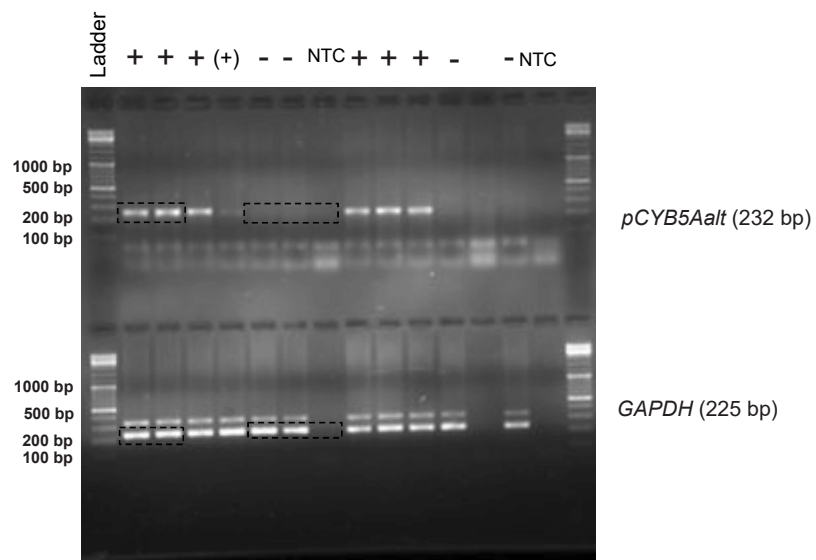

Supplement: Supplementary file 17 — Additional file 17. Uncropped electrophoretic gel of Fig. 3. A RT-PCR was used to confirm overexpression of pCYB5Aalt in NALM-6 cell line. Uncropped electrophoretic gel (agarose, 1.6%) of RT-PCR results of Fig. 3 are shown. Cells were transfected with pCYB5Aalt (+) or pEmpty (−). GAPDH was used as control. NTC = non-template-control. Size of PCR bands are shown in bp. Cropped parts of gel that were used for Fig. 3 are indicated by dotted, black rectangles. [file 12863_2022_1041_MOESM17_ESM.pdf]
