# Supplementary material for: An alternative CYB5A transcript is expressed in aneuploid ALL and enriched in relapse
Source: BMC Genom Data. 2022 Apr 18;23:30. doi: 10.1186/s12863-022-01041-1 (PMC9014596; doi:10.1186/s12863-022-01041-1)
Supplement: Supplementary file 14 — Additional file 14. Expression of BH3-motif genes in engineered cell lines and combined cohort 3. BH3-motif genes are subdivided according to their reported roles in apoptosis in pro-apoptotic (BAD, BID, BCL2L11), anti-apoptotic (BCL2, BCL2L2, BCL2A1) and isoform-dependent (MCL1, BCL2L1). A BH3-motif gene expression (in TPM) is compared between pCYB5Aalt NALM-6 (n = 3) and empty vector controls (n = 2). Dots represent expression of individual clones. B Gene expression is compared between samples positive for CYB5Aalt (n = 22) and negative samples (n = 198) in combined cohort 3 (n = 220). Dots represent mean expression; vertical lines show standard error of mean. FDR = false discovery rate (Benjamini-Hochberg method). [file 12863_2022_1041_MOESM14_ESM.pdf]

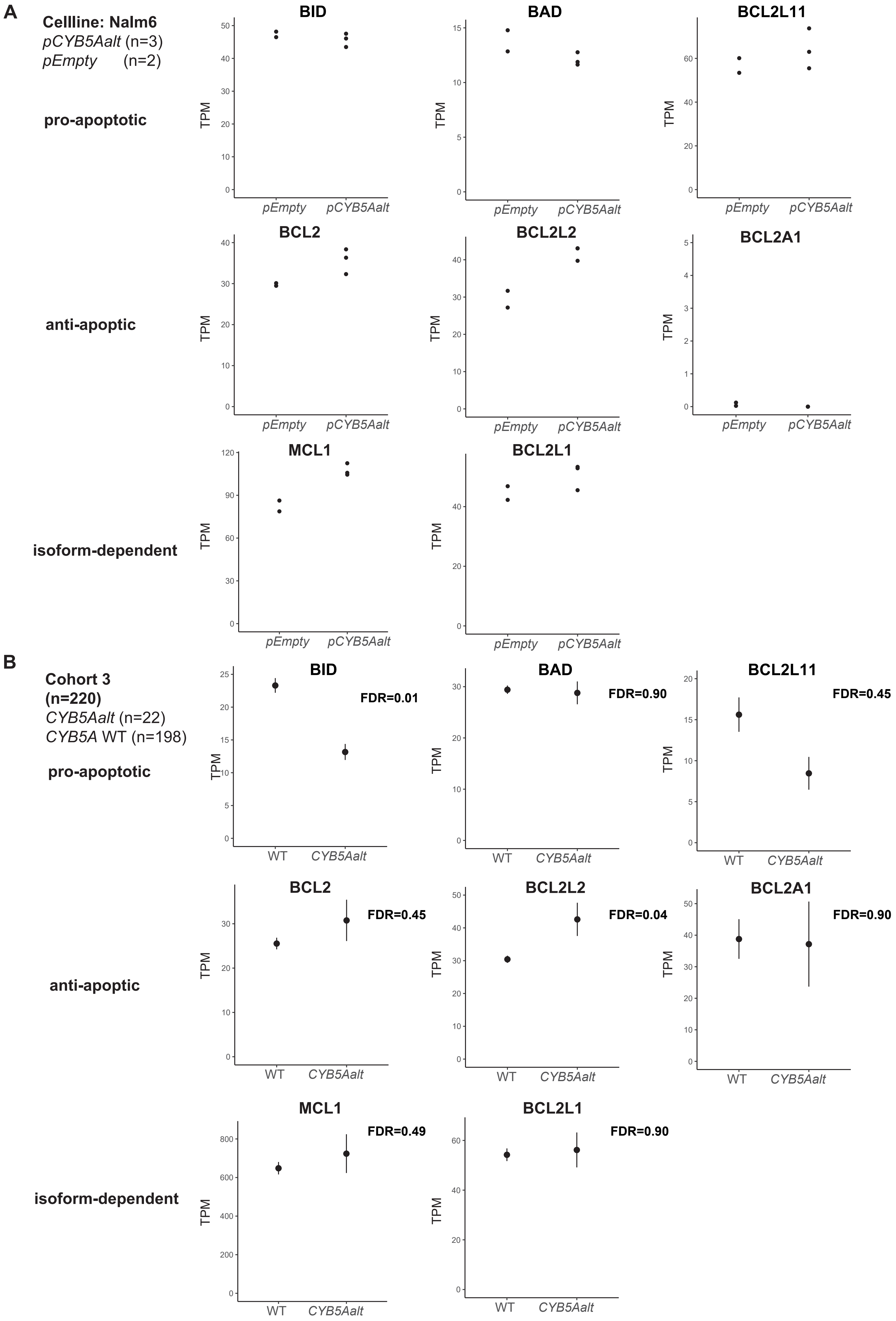
A
Cellline: Nalm6
pCYB5Aalt (n=3)
pEmpty (n=2)
pro-apoptotic
anti-apoptic
isoform-dependent
BID
BAD
BCL2L11
BCL2
BCL2L2
BCL2A1
MCL1
BCL2L1
TPM
pEmpty
pCYB5Aalt
B
Cohort 3
(n=220)
CYB5Aalt (n=22)
CYB5A WT (n=198)
pro-apoptotic
anti-apoptic
isoform-dependent
BID
FDR=0.01
BAD
FDR=0.90
BCL2L11
FDR=0.45
BCL2
FDR=0.45
BCL2L2
FDR=0.04
BCL2A1
FDR=0.90
MCL1
FDR=0.49
BCL2L1
FDR=0.90
TPM
WT
CYB5Aalt
